# Producing treatment hierarchies in network meta-analysis using probabilistic models and treatment-choice criteria

**DOI:** 10.1017/rsm.2026.10071

**Published:** 2026-02-20

**Authors:** Theodoros Evrenoglou, Adriani Nikolakopoulou, Guido Schwarzer, Gerta Rücker, Anna Chaimani

**Affiliations:** 1 Institute of Medical Biometry and Statistics, Faculty of Medicine and Medical Center-University of Freiburg, Freiburg im Breisgau, Germany; 2 Center of Research in Epidemiology and Statistics (CRESS-U1153), Université Paris Cité, INSERM, Paris, France; 3 Department of Hygiene, Social-Preventive Medicine and Medical Statistics, School of Medicine, Aristotle University of Thessaloniki, Thessaloniki, Greece; 4 Oslo Center for Biostatistics and Epidemiology, Department of Biostatistics, University of Oslo, Oslo, Norway

**Keywords:** clinically important difference, network of interventions, probabilistic regression model, smallest worthwhile difference, treatment hierarchy

## Abstract

A key output of network meta-analysis (NMA) is the relative ranking of treatments; nevertheless, it has attracted substantial criticism. Existing ranking methods often lack clear interpretability and fail to adequately account for uncertainty, overemphasizing small differences in treatment effects. We propose a novel framework to estimate treatment hierarchies in NMA using a probabilistic model, focusing on a clinically relevant treatment-choice criterion (TCC). Initially, we define a TCC based on smallest worthwhile differences (SWD), converting NMA relative treatment effects into treatment preference format. These data are then synthesized using a probabilistic ranking model, assigning each treatment a latent “ability” parameter, representing its propensity to yield clinically important and beneficial true treatment effects relative to the rest of the treatments in the network. Parameter estimation relies on the maximum likelihood theory, with standard errors derived asymptotically from the Hessian matrix. To facilitate the use of our methods, we launched the R package mtrank. We applied our method to two clinical datasets: one comparing 18 antidepressants for major depression and another comparing 6 antihypertensives for the incidence of diabetes. Our approach provided robust, interpretable treatment hierarchies that account for a concrete TCC. We further examined the agreement between the proposed method and existing ranking metrics in 153 published networks, concluding that the degree of agreement depends on the precision of the NMA estimates. Our framework offers a valuable alternative for NMA treatment ranking, mitigating overinterpretation of minor differences. This enables more reliable and clinically meaningful treatment hierarchies.

## Highlights

### What is already known?

The relative ranking of different competing treatments is a key output of a network meta-analysis (NMA). However, ranking has been widely criticized for being prone to overinterpretation and for overemphasizing small differences in treatment effects. In addition, current ranking metrics lack a straightforward way to measure uncertainty in treatment rankings. This creates challenges in interpretation, particularly when treatments are ranked adjacently.

### What is new?

We introduce a new framework for estimating treatment hierarchies in network meta-analysis (NMA) using a probabilistic model, focusing on a clinically relevant treatment-choice criterion (TCC). This TCC is mathematically defined using the smallest worthwhile difference (SWD), which represents the smallest beneficial effect of a treatment that justifies a preference for it over another. We apply this TCC to all NMA estimates to determine either a treatment preference or a tie. The treatment preferences are synthesized using a probabilistic ranking model, where each treatment is assigned a latent “ability” parameter that reflects its propensity to yield clinically important and beneficial treatment effects, according to the TCC, when compared to other treatments in the network. In this way, treatments with higher estimated ability occupy a higher position in the final ranking list. The ability-based ranking metric is estimated via maximum likelihood, and standard errors are derived asymptotically from the Hessian matrix. To support the practical application of our method, we have developed and released the R package mtrank.

### Potential impact for RSM readers

Our framework provides a new way to estimate treatment hierarchies in NMA, offering an alternative to existing ranking methods. Recent studies stress the importance of clearly defining treatment hierarchy questions before estimating rankings. To our knowledge, this is the first method to explicitly and quantitatively address this question using a predefined TCC. Researchers can use this method as a primary ranking tool or as a sensitivity analysis alongside traditional metrics, especially when clinically relevant TCCs are known.

## 1 Introduction

Interpretation of network meta-analysis (NMA) outputs can be challenging as it usually comprises consideration of multiple treatment effects with different levels of uncertainty and credibility across comparisons in the network.^1^^,^ ^2^ For example, in the relatively simple case of a network with 6 treatments the output of NMA consists of 15 treatment effect estimates. In such a context, treatment ranking can be a reliable way to summarize the evidence provided by complex treatment networks.^1^^,^ ^3^^,^ ^4^ This may explain the fact that treatment hierarchies are frequently presented in published NMAs with 43% of them reporting at least one ranking metric.^5^

Probably the most commonly used ranking metric, until recently, was the probability of a treatment to have the best value,^5^ usually denoted as $p_{BV}$ . This is primarily a Bayesian metric, but it can also be calculated within the frequentist framework using resampling, thereby mimicking a Bayesian framework with flat priors. It represents the probability that a treatment in the network will have the best true treatment effect.^6^ Although $p_{BV}$ has been widely used in published NMAs, more recently it has been criticized for not properly accounting for the uncertainty of the NMA estimates.^4^^,^ ^7^^,^ ^8^

Other common ranking metrics are P-scores ,^4^ which are obtained analytically through the cumulative density function of the standard normal distribution, or their Bayesian equivalent SUCRA^1^ that represent the surface under the cumulative ranking curve for each treatment. The main limitation of these metrics is that they often lead to attributing distinct ranks to treatments even when there are only small differences between their SUCRA values or P-scores. Nikolakopoulou et al.^7^ employed the “deviation from the means” approach for the construction of the design matrix in the NMA model and introduced a new ranking metric, called the probability of a treatment being preferable to a fictional treatment of average performance (PReTA). This metric potentially accounts better for the uncertainty in the relative effects than P-scores or SUCRAs, particularly when there is substantial variability in the precision of the NMA estimates. This is an important advantage since an empirical study revealed high agreement across all ranking metrics when NMA estimates had similar variance estimates, but large sensitivity to the choice of metric for networks with large discrepancies in the variance of the NMA estimates.^5^ More recently, new ranking metrics and approaches have been developed to address more complex ranking questions. Mavridis et al.^9^ extended P-scores to incorporate clinically important values, while Curteis et al. proposed a similar extension in terms of the SUCRA ranking method.^10^ Chaimani et al.^11^ suggested that treatment rankings should consider not only the summary relative effects but also other information, such as study or treatment characteristics. They introduced a new metric, called the probability of selecting a treatment to recommend (POST-R) that implements additional characteristics in treatment hierarchy (e.g., risk of bias or treatment cost). Papakonstantinou et al.^12^ developed a resampling approach for estimating the probability that a specific treatment hierarchy occurs or a predefined criterion may be met.

Despite its usefulness when properly reported and interpreted, treatment ranking in NMA has been accompanied with a lot of skepticism.^13^^–^ ^16^ Other common arguments against treatment ranking include that it can be biased, it is difficult to interpret, it is not accompanied with uncertainty measures, and it may overemphasize nonimportant differences in the treatment effect estimates.^14^^,^ ^15^ For example, Kibret et al. performed a simulation study and found that ranking can be biased when there is an unequal number of studies per comparison in the network, with the rank probability for the treatment included in the fewest number of studies tending to suffer from upward bias.^16^ However, Salanti et al.^6^ argued that these criticisms should not refer to the ranking metrics per se but to the way they are used and interpreted. This is because different metrics target different types of hierarchy questions and researchers should clearly define what they mean by “best treatment” in a given setting. Hence, setting a well-defined treatment hierarchy question should always precede the estimation of treatment ranking and drive the choice of the ranking metric.^6^

In this article, we introduce a novel approach for estimating treatment hierarchies in NMA based on a treatment-choice criterion (TCC) constructed to ensure clinically important treatment effects. This TCC splits the NMA estimates into those that fulfil the criterion indicating a treatment effect that justifies a treatment preference and those that do not indicate a clear treatment preference. We then use a probabilistic model that yields the final treatment hierarchy by synthesizing the treatment preferences obtained from the TCC. Our manuscript is organized as follows. First, we define the TCC based on clinically important values. We then apply the criterion to the NMA treatment effects, taking into account their confidence intervals to get either a treatment preference or a tie. Our synthesis model estimates the treatment hierarchy through a latent parameter assigned to each treatment in the network that represents its “ability” to yield clinically important and beneficial treatment effects in context of the defined TCC. In this way, treatments with higher estimated abilities are positioned more prominently in the final ranking. This modeling approach has been previously used to produce rankings in fields outside of medicine, such as sports science,^17^ animal behavior,^18^ and risk analysis.^19^ To illustrate our method and compare it with existing alternatives, we use two published NMAs: one comparing different antidepressants^20^ for major depression and a second evaluating different antihypertensives^21^ for the incidence of diabetes. Finally, we investigate the agreement between the new and existing ranking metrics through an empirical study where we reanalyze 153 published networks.^22^^,^ ^23^

## 2 Methods

### 2.1 Defining treatment-choice criteria based on NMA estimates

Suppose a network of N studies comparing T treatments. Let $\hat{\theta }={\left[{\hat{\theta }}_{XY}\right]}_{X\neq Y},\mathrm{where}\;X,Y\in \left\{1,2,\ldots ,T\right\}$ , denote the (T2) -vector containing all treatment effect estimates obtained from the NMA. Let also $l={\left[l_{XY}\right]}_{X\neq Y}$ and $u={\left[u_{XY}\right]}_{X\neq Y},X,Y\in \left\{1,2,\ldots ,T\right\}$ represent the corresponding vectors containing the lower and upper bounds of the confidence intervals for each ${\hat{\theta }}_{XY}$ . We start building our modeling approach by defining concrete criteria for choosing one treatment over another or considering two treatments as equivalent. These criteria have the form of a decision rule and may depend on several factors, such as the clinical setting, the outcome(s) under investigation, or even the type of patients under consideration (e.g., chronic patients vs. treatment-naïve individuals). Here, we suggest a generic approach that can be easily adapted to different settings based on the so-called range of equivalence (ROE). The ROE has been previously introduced as a way to infer on the clinical importance of a treatment effect in the context of appraising NMA estimates; relative effects lying within this range are considered lacking a treatment preference.^24^

**Figure 1:**
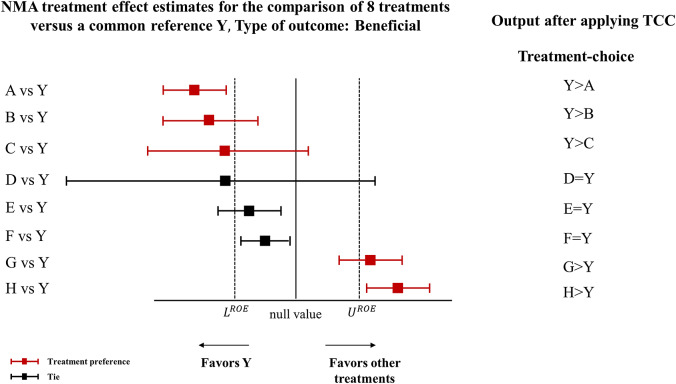
A graphical representation of the TCC for a fictional example showing the NMA estimates for the comparison of eight treatments versus a common reference treatment Y in terms of a beneficial outcome. Figure 1 Long description.

Following Nikolakopoulou et al.,^24^ we construct the ROE using the smallest worthwhile difference (SWD), representing the smallest beneficial effect of a treatment that justifies a preference for it over another treatment,^25^ and its reciprocal (or opposite) value. For example, for a given SWD equal to 1.25 on the odds ratio (OR) scale, the ROE would range from $\frac{1}{1.25}$ to 1.25. Based on the ROE, the TCC distinguishes between treatment preferences or ties as follows: a comparison between treatments X and Y will indicate no clear treatment preference when either ${\hat{\theta }}_{XY}$ lies within ROE or its confidence interval bounds $l_{XY}$ and $u_{XY}$ extend in opposite directions beyond the ROE. In such cases, the TCC is not satisfied, as there is insufficient evidence to support a clear treatment preference, and therefore treatments X and Y are considered as equivalent (i.e., X=Y) . In all other cases, the TCC is fulfilled and a treatment preference is defined (i.e., either X>Y or X<Y ) based on the direction of the effect ${\hat{\theta }}_{XY}$ . Figure 1 illustrates the above TCC for the case of a beneficial outcome (i.e., larger treatment effect values are desirable) in a fictional example. The mathematical representation of this rule is available in our Supplementary Material.

All NMA estimates are translated into a treatment preference format by applying the TCC, indicating either a treatment preference or a tie. Throughout the remainder of this manuscript, treatment effects that fulfil the TCC and therefore justify a treatment preference are also referred to as clinically important effects. Note that the TCC, as defined here, serves as a generic decision rule. Although not formally grounded in mathematical principles, the described TCC has been widely adopted by other established frameworks, such as CINeMA^24^^,^ ^26^ and GRADE,^27^^–^ ^29^ which aim to put NMA estimates into a decision-analytic framework. In practical scenarios, investigators may also adjust this TCC based on their context specific needs. A crucial step in defining the TCC in our setting involves determining the SWD which is always context specific. To this end, both statistical and elicitation-based approaches for defining a SWD have been suggested elsewhere and therefore this task is beyond the scope of this article.^25^^,^ ^30^^–^ ^33^

### 2.2 Estimating treatment hierarchies based on treatment-choice criteria using probabilistic models

To synthesize the treatment preferences obtained from the TCC, we adapt the so-called “Bradley–Terry model”^33^^–^ ^36^ to the context of NMA. This is a probabilistic model, suitable for modeling preference data and originally suggested to estimate ranking outside NMA (e.g., sports tournaments) but, to the best of our knowledge, was never adapted to estimate treatment hierarchies in NMA.

We parameterize the model using an unobserved latent parameter $\psi_{X}\geq 0$ that represent the “ability” each treatment X has to outperform the other treatments in the network conditional on the TCC. In this context, the term “ability to outperform” refers to the propensity of a treatment X to produce clinically important and beneficial true treatment effects, as determined from the TCC, relative to the remaining treatments in the network. Throughout the manuscript, the term “outperform” will be used according to this definition. Given all these considerations, the treatment hierarchy question addressed here is “Based on the predefined TCC, what is the overall propensity of each treatment to yield clinically important and beneficial true treatment effects when compared to the rest of the treatments in the network?” Consequently, the true ability of each treatment is the parameter of interest here, modeled through a Bradley–Terry model.^33^^–^ ^36^

Establishing a direct one-to-one mathematical relationship between the true treatment effects and the treatment abilities is challenging as the former are fixed unknown parameters, while the latter are unobserved treatment characteristics that depend jointly on (i) the magnitude of the true treatment effects and (ii) the TCC of interest, defining clinical important effects. Nevertheless, it is expected that treatments associated with estimated treatment effects fulfilling the TCC will yield larger ability estimates. In this way, treatments with higher estimated ability estimates will occupy higher positions in the final ranking list.^34^ Let also ψ denote the T -length vector that contains the ability of each treatment in the network.

The idea behind this model stems from Luce’s axiom^34^^,^ ^37^^,^ ^38^ of choice which states that the probability that a treatment X has the largest ability among all T treatments, with respect to the TCC, is equal to $\frac{\psi_{X}}{\sum_{i=1}^{T}\psi_{i}}$ . Luce’s axiom of choice is valid under the assumption of independence of irrelevant alternatives. In the NMA setting, this assumption states that whether treatment X is ranked higher than treatment Y does not depend on other treatment options. This assumption is expected to hold whenever the underlying NMA assumption of transitivity holds, as in such cases the true treatment effect $\theta_{XY}$ is consistent across both direct and indirect comparisons.

#### 2.2.1 Synthesizing treatment preferences obtained from the treatment-choice criterion

Following the earlier axiom, for each pairwise comparison in the network, the probability that treatment X will outperform treatment Y ( X≠Y;X,Y,=1,2,…,T ) is

(1) $$\begin{array}{r} \mathrm{Pr}\left(X>Y\right)=\frac{\psi_{X}}{\psi_{X}+\psi_{Y}} \end{array}$$

with $\psi_{X}\geq 0\;\forall X\in \left\{1,2,\ldots ,T\right\}$ and $\sum_{i=1}^{T}\psi_{i}=1$ . Based on Equation (1), a logit-linear Bradley–Terry model can be parametrized as

(2) $$\begin{array}{r} logit(Pr(X>Y))=\log(\psi_{X})-\log(\psi_{Y}) \end{array}$$

Equation (1) requires that one treatment is always preferred over another for any pairwise comparison in the network. However, this can violate the TCC defined in Section 2.1 where we also consider that two treatments may not justify a treatment preference. To accommodate for ties (i.e., Pr(X=Y)) , following Davidson,^37^ we assume that the probability of a tie between two treatments X and Y relates to $\nu \sqrt{\psi_{X}\psi_{Y}}$ . The quantity $\sqrt{\psi_{X}\psi_{Y}}$ is the geometric mean of $\psi_{X}$ and $\psi_{Y}$ , while ν is a scalar nuisance parameter that describes the prevalence of ties in the network. Hence, the probability that X outperforms Y now becomes

(3) $$\begin{array}{r} \mathrm{Pr}\left(X>Y\right)=\frac{\psi_{X}}{\psi_{X}+\psi_{Y}+\nu \sqrt{\psi_{X}\psi_{Y}}} \end{array}$$

and the probability that the two treatments are tied is

(4) $$\begin{array}{r} \mathrm{Pr}\left(X=Y\right)=\frac{\nu \sqrt{\psi_{X}\psi_{Y}}}{\psi_{X}+\psi_{Y}+\nu \sqrt{\psi_{X}\psi_{Y}}} \end{array}$$

with $\psi_{X}\geq 0,\forall X\in \left\{1,2,\ldots ,T\right\},\nu >0$ and $\sum_{i=1}^{T}\psi_{i}=1$ . Note that parametrizing the probability of a tie using Equation (4) offers the mathematical convenience that, for a fixed value of ν , the probability of a tie is maximized when $\psi_{X}=\psi_{Y}$ . In other words, the probability of a tie depends only on the ratio of $\psi_{X}$ and $\psi_{Y}$ and is maximized between treatments with equal abilities. The mathematical proof of this is provided in the Supplementary Material. In this manuscript, the estimation process for the earlier model refers to the frequentist framework and relies on maximum likelihood theory.^33^^,^ ^39^ Fitting the model in the Bayesian setting is also possible and has been discussed elsewhere.^40^^,^ ^41^

Let $r_{XY}$ denote a variable that takes the value 1 if, based on the TCC, treatment X is preferred over treatment Y and 0 otherwise. Let also $w_{XY}$ be the tie variable that takes the value 1 if the TCC indicates that X=Y ; otherwise it is equal to 0. Then, the log-likelihood function for the model described in Equations (3) and (4) can be written as

(5) $$\begin{array}{rl} L(\psi ,\nu )= & \;\sum \sum_{X\neq Y}r_{XY}\log\;\left(\frac{\psi_{X}}{\psi_{X}+\psi_{Y}+\nu \sqrt{\psi_{X}\psi_{Y}}}\right)\\ & +r_{YX}\log\;\left(\frac{\psi_{Y}}{\psi_{X}+\psi_{Y}+\nu \sqrt{\psi_{X}\psi_{Y}}}\right)\\ & +w_{XY}\log\;\left(\frac{\nu \sqrt{\psi_{X}\psi_{Y}}}{\psi_{X}+\psi_{Y}+\nu \sqrt{\psi_{X}\psi_{Y}}}\right) \end{array}$$

with $\sum_{i=1}^{T}\psi_{i}=1$ and ν>0 . Maximizing the multinomial log-likelihood in Equation (5) yields the MLEs of the ability parameters ψ . The asymptotic distribution of $\hat{\psi }$ is a multivariate normal distribution with mean ψ and variance–covariance matrix $\Sigma^{-1}$ obtained as the inverse of the Hessian matrix Σ . The elements of Σ correspond to the second partial derivatives of the log-likelihood in Equation (5). Finally, note that Equation (5) can yield ability estimates $\hat{\psi }$ only when treatment preferences are identified from the TCC. In other words, the proposed methodology cannot estimate any treatment hierarchy if only ties, thus no clinically important NMA estimates, are obtained from the TCC.

#### 2.2.2 Absolute and relative treatment abilities

Maximizing Equation (5) in terms of ψ refers to an optimization problem constrained at the region $\left\{\psi_{X}\geq 0,\sum_{i=1}^{T}\psi_{i}=1\right\}$ . This constraint prevents from negative estimates of the ability parameters and guarantees that the optimization problem remains identifiable. Then, the resulting ${\hat{\psi }}_{X}$ represents the estimated absolute abilities of each treatment in the network. However, as also noted elsewhere,^34^ the scale of the absolute ability estimates is immaterial; what matters here is the relative comparison between abilities. To address this issue, we construct an artificial reference treatment group^35^ T+1 , with ability equal to the average of the absolute ability estimates across all the T treatments. This implies that we assume the ability of the treatment T+1 being equal to $\psi_{T+1}=\frac{\sum_{i=1}^{T}{\hat{\psi }}_{i}}{T}$ . Then, the ranking results are presented in terms of the ability ratios $\frac{{\hat{\psi }}_{X}}{\psi_{T+1}}\forall X\in \left\{1,2,\ldots ,T\right\}$ .

The final estimates ${\hat{\psi }}_{X}$ do not necessarily satisfy $\sum_{i=1}^{T}\psi_{i}=1$ , as the renormalization of the vector ψ is not needed after each iteration of the iterative process.^39^ However, based on Luce’s axiom of choice,^37^^,^ ^38^ we can renormalize the absolute ability estimates as ${\hat{\pi }}_{X}=\frac{{\hat{\psi }}_{X}\;}{\sum_{i=1}^{T}{\hat{\psi }}_{i}\;}$ . This allows interpreting ${\hat{\pi }}_{X}$ as the probability that each treatment X∈{1,2,…,T} has the largest true ability to yield clinically important and beneficial treatment effects, with respect to the TCC, among all the T treatments in the network. This additional probabilistic ranking metric, ${\hat{\pi }}_{X}$ , offers a straightforward interpretation, but it does not account for the uncertainty of the ability estimates ${\hat{\psi }}_{X}.$ Therefore, we propose ${\hat{\pi }}_{X}$ be presented alongside the ability estimates ${\hat{\psi }}_{X}$ , particularly when these estimates are derived with similar levels of uncertainty in the top positions of the ranking list.

## 3 A qualitative comparison between the new ranking metric and other existing approaches

Table 1 summarizes the principal similarities and differences between the newly proposed and existing common ranking metrics, each addressing a different treatment hierarchy question. The interpretation of results obtained from our new method does not directly allow for probabilistic statements, in contrast to the existing ranking metrics that yield inherently probabilistic quantities and thus permit a more straightforward interpretation. Within the framework of the proposed approach, the scale of the ability estimates is irrelevant; the interpretation of ${\hat{\psi }}_{X}$ arises solely from its relative comparison with the corresponding ability estimates of other treatments in the network.

**Table 1:** A summary of the characteristics across different ranking methods Table 1 Long description.

	Proposed ability-based metric ( ${\hat{\psi }}_{X}$ )	P-scores (or PReTA)	$p_{BV}$	P-scores (SWD)
What is the treatment hierarchy question?	Based on the predefined TCC, what is the overall propensity of treatment X to yield clinically important and beneficial true treatment effects when compared to the rest of the treatments in the network?	What is the proportion of treatments in the network that are inferior to treatment X ?	What is the probability that each treatment yields the largest true treatment effect?	What is the proportion of treatments in the network that are inferior to treatment X by a margin at least equal to SWD?
How results are interpreted?	Ability estimate ${\hat{\psi }}_{X}$ measures the overall propensity of treatment X to fulfil the TCC, and, thus to yield clinically important and beneficial true effects when compared to the rest of the treatments The absolute scale of the ability estimate ${\hat{\psi }}_{X}$ is irrelevant. Interpretation stems from the relative comparison of ${\hat{\psi }}_{X}$ with the rest ability estimates	Value for treatment X expresses the proportion of treatments worse than X	Value for treatment X expresses the probability that X yield the largest true effect	Value for treatment X expresses the proportion of treatments worse than X by a margin of SWD
Does it account for clinical importance?	Yes, a TCC constructed based on clinical important values is applied to the NMA estimates. Then, its output is synthesized via a Bradley–Terry model	No	No	Yes, it relies on the probability that NMA estimates are larger than SWD
Impact of clinical importance in the hierarchy	Across different SWD values, and thus different TCC, differences in the treatment hierarchy can occur	–	–	Treatment hierarchy does not change across different SWD values P-scores and P-scores (SWD) always yield the same hierarchy
Availability of ranking	When only ties are identified by the TCC the method does not yield a treatment hierarchy	A treatment hierarchy is always available	A treatment hierarchy is always available	A treatment hierarchy is always available

A probabilistic interpretation can, however, be derived through the normalized abilities ${\hat{\pi }}_{X}$ , which estimate the probability that each treatment has the largest true ability under the prespecified TCC. Since ${\hat{\pi }}_{X}$ does not incorporate the uncertainty associated with ${\hat{\psi }}_{X}$ , its use is recommended as a supplementary rather than a primary metric which in the context of the proposed method should always be ${\hat{\psi }}_{X}$ . It is further noted that the interpretation of ${\hat{\pi }}_{X}$ is analogous, though not equivalent, to that of $p_{BV}$ : while ${\hat{\pi }}_{X}$ pertains to the probability that treatment X exhibits the greatest true ability under the defined TCC, $p_{BV}$ relates to the underlying true treatment effects without incorporating a TCC. For example, a treatment X yielding large yet clinically unimportant effects may attain a high $p_{BV}$ but a low ${\hat{\pi }}_{X}$ .

An additional distinction between the proposed method and other ranking approaches lies in whether, and how, they account for the clinical importance of the NMA estimates and the extent to which this consideration influences the resulting treatment hierarchy. The proposed method explicitly distinguishes clinically important from negligible NMA treatment effects by applying a predefined TCC to the NMA estimates. Subsequently it estimates ${\hat{\psi }}_{X}$ as a measure of the overall propensity of treatment X to fulfil the TCC when compared to the rest of the treatments in the network. In the context of the TCC described in Section 2.1, the choice of the SWD can affect the final treatment hierarchy, as the propensity of each treatment to fulfil the TCC may vary depending on the selected SWD. For instance, a treatment with modest efficacy may yield treatment effects that satisfy the TCC for small SWD values, but fail to do so if larger thresholds are deemed clinically meaningful in the decision-making process.

Clinical importance is treated differently within the P-scores (SWD) framework, where the SWD is not used to define a TCC but is instead applied directly to the NMA estimates. This approach is based on the probabilities that the NMA estimates exceed the SWD, and consequently, variations in the SWD influence these probabilities only numerically, without altering the resulting treatment hierarchy. The same holds when comparing the hierarchy obtained from the standard P-scores approach, which does not incorporate clinical importance, with that derived from the P-scores (SWD) method as both of these methods are expected to always yield the same treatment hierarchy.

The final principal distinction among the different methods concerns the availability of the ranking list. The newly proposed method does not produce a treatment hierarchy when only ties are identified based on the TCC. This feature relates only to the new approach and indicates that a treatment ranking cannot be established in the absence of clinically important NMA estimates. In contrast, the remaining ranking metrics always yield a treatment hierarchy. Although these approaches do not explicitly incorporate a TCC in their computation, we recommend that meta-analysts interpret ranking with caution when clinically important NMA estimates are lacking.

## 4 Applications

We illustrate the use of our treatment ranking method and compare it with existing ranking approaches using two published networks. The first compares the efficacy of several antidepressants for major depression^20^ and the second compares different antihypertensive treatment classes and placebo for the incidence of diabetes.^21^ We compared five ranking approaches: (a) P-scores,^4^ (b) P-scores “adjusted” for the SWD,^9^ (c) the PReTA-ranking,^7^ (d) the ranking according to $p_{BV}$ in the frequentist setting, and (e) the estimated treatment abilities from our ranking approach. All ranking metrics were calculated based on a random-effects NMA model. To conduct the analysis, we used R version 4.4.1 (2024-06-14) and we used the R package netmeta^42^ to fit the NMA models. To facilitate the use of our proposed approach, we have created the R package mtrank^43^ which is available on CRAN.

### 4.1 Antidepressants for major depression

**Figure 2:**
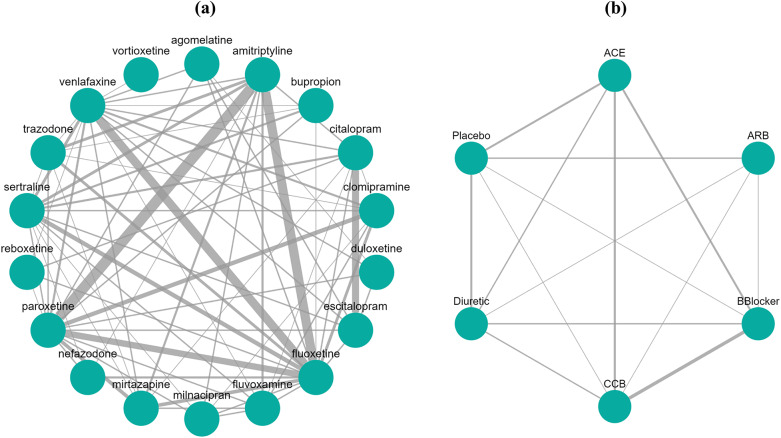
Network plots for the two clinical examples. (a) The network of antidepressants and (b) the network of antihypertensive treatments. ACE, angiotensin-converting enzyme inhibitors; ARB, angiotensin receptor blockers; CCB, calcium channel blocker; BBlocker, beta blocker. Figure 2 Long description.

This network comprises 179 trials comparing 18 antidepressant drugs (Figure 2a). The primary outcome is response to treatment defined as a 50% or greater reduction in a depression symptom scale between baseline and 8 weeks of follow-up. The outcome is measured as odds ratios (OR).

The results for methods (a) to (d) are presented in Table 2, alongside the respective NMA estimates of all treatments versus Trazodone. In this network, large treatment effect values indicate beneficial effects. A consensus is observed in terms of the best treatment for the P-scores and the $p_{BV}$ which rank Vortioxetine first, while using the PReTA-ranking Escitalopram is placed at the first position and Vortioxetine second. Results in terms of median ranks are available in Table 1 in the Supplementary Material. These results show that Vortioxetine, Escitalopram, and Bupropion occupy the top three positions, though there is considerable uncertainty. The median ranks and 95% CIs were 1 [1, 15], 3 [1, 10], and 3 [1, 15], respectively.

**Table 2:** Ranking metrics for the network of antidepressants. Treatments with the top three values for each respective metric are shown in bold. The “Treatment” column is ordered according to P-scores Table 2 Long description.

Treatment	Odds ratio of treatment vs. placebo [position in ranking]	P-scores	P-scores (SWD)	PReTA	$p_{BV}$	${\hat{\pi }}_{X}$
Vortioxetine	**1.87 [1]**	**0.90**	**0.75**	**0.93**	**0.64**	**0.20**
Escitalopram	**1.51 [3]**	**0.83**	**0.49**	**0.98**	**0.07**	**0.36**
Bupropion	**1.55 [2]**	**0.79**	**0.53**	0.87	**0.19**	**0.20**
Mirtazapine	1.44 [4]	0.75	0.39	**0.91**	0.03	0.06
Amitriptyline	1.40 [5]	0.71	0.33	0.88	0.01	0.06
Agomelatine	1.36 [6]	0.64	0.29	0.74	0.01	0.06
Paroxetine	1.34 [7]	0.62	0.25	0.83	0.00	0.01
Venlafaxine	1.34 [8]	0.61	0.25	0.78	0.00	0.01
Duloxetine	1.29 [9]	0.52	0.21	0.53	0.01	0.01
Milnacipran	1.27 [10]	0.49	0.19	0.46	0.01	0.01
Sertraline	1.25 [11]	0.45	0.15	0.38	0.00	0.00
Nefazodone	1.18 [13]	0.38	0.16	0.33	0.02	0.00
Citalopram	1.20 [12]	0.37	0.12	0.24	0.00	0.00
Clomipramine	1.13 [15]	0.26	0.07	0.10	0.00	0.00
Fluvoxamine	1.12 [16]	0.25	0.07	0.10	0.00	0.00
Fluoxetine	1.13 [14]	0.23	0.06	0.01	0.00	0.00
Trazodone	1.00 [17]	0.12	0.03	0.02	0.00	0.00
Reboxetine	0.95 [18]	0.09	0.02	0.02	0.00	0.00

**Figure 3:**
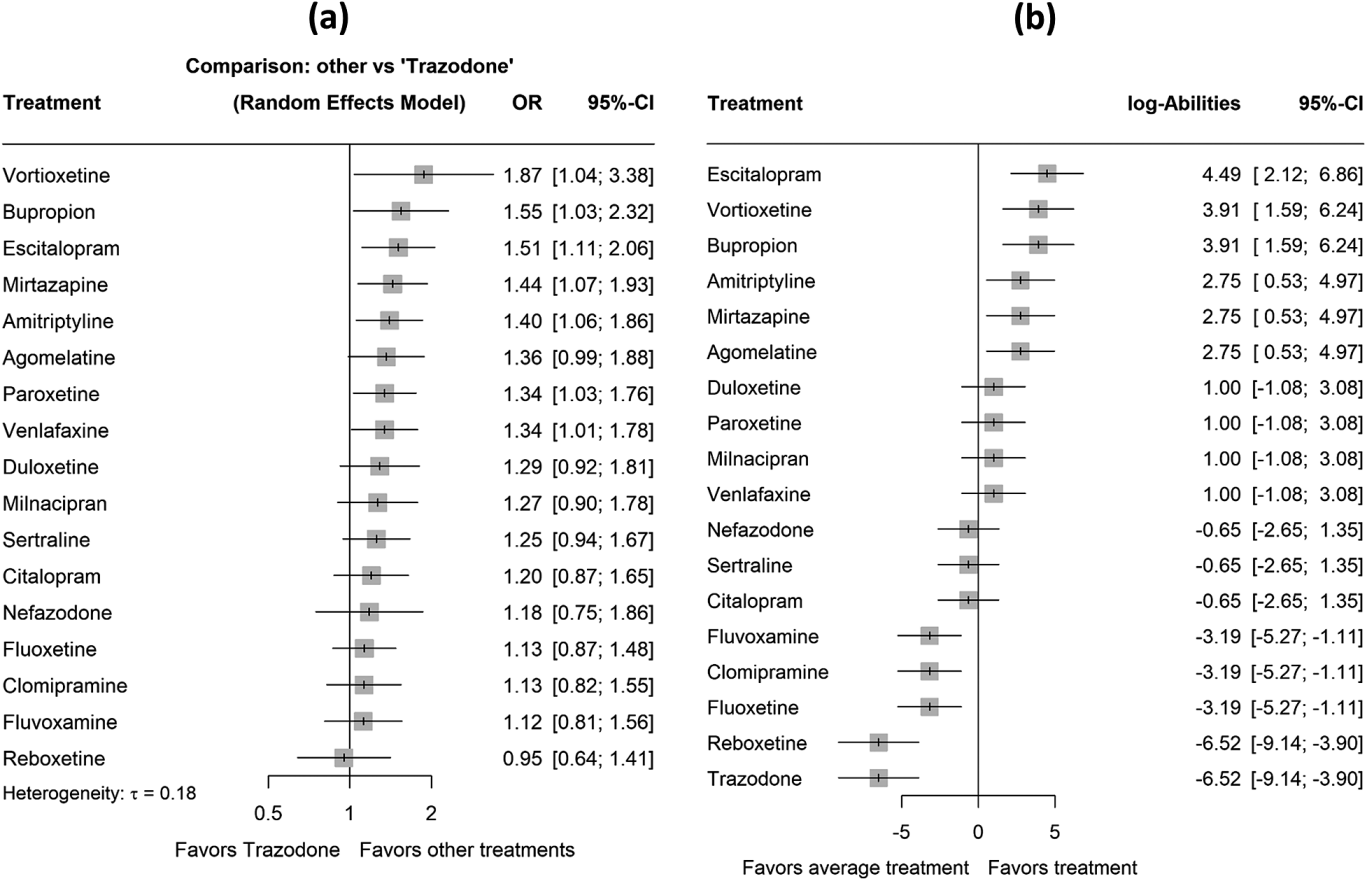
Forest plots with results for the network of antidepressants. (a) The summary odds ratios obtained assuming Trazodone as the reference treatment group. (b) The ranking results obtained using the proposed methodology. Figure 3 Long description.

The NMA treatment effect estimates for the comparison of each treatment versus Trazodone are also shown in Figure 3a. Overall, all NMA treatment effect estimates favor the other treatments over Trazodone. Vortioxetine has the largest treatment effect and ranks first, but it also has the largest standard error. When using $p_{BV}$ , the ranking does not fully account for the uncertainty in treatment effect estimates. This explains why Vortioxetine appears to be clearly the best treatment according to $p_{BV}$ .

Following the original publication,^20^ we assume a SWD equal to 1.20. Using SWD adjusted P-scores, Vortioxetine was ranked at the top position and clearly higher than Bupropion which is at the second position. The differences between unadjusted and SWD adjusted P-scores can be attributed to the increased emphasis that the latter approach puts on the magnitude of the NMA estimates. Note that the adjusted P-scores approach affects only the numerical values of the unadjusted P-scores and is generally not expected to alter the treatment hierarchy. Overall, the differences across the different hierarchies may be explained by the substantial variation of the standard errors across the NMA estimates that range from 0.07 to 0.33. The full distribution of the standard errors across all NMA estimates is depicted in Figure 1 in the Supplementary Material.

**Figure 4:**
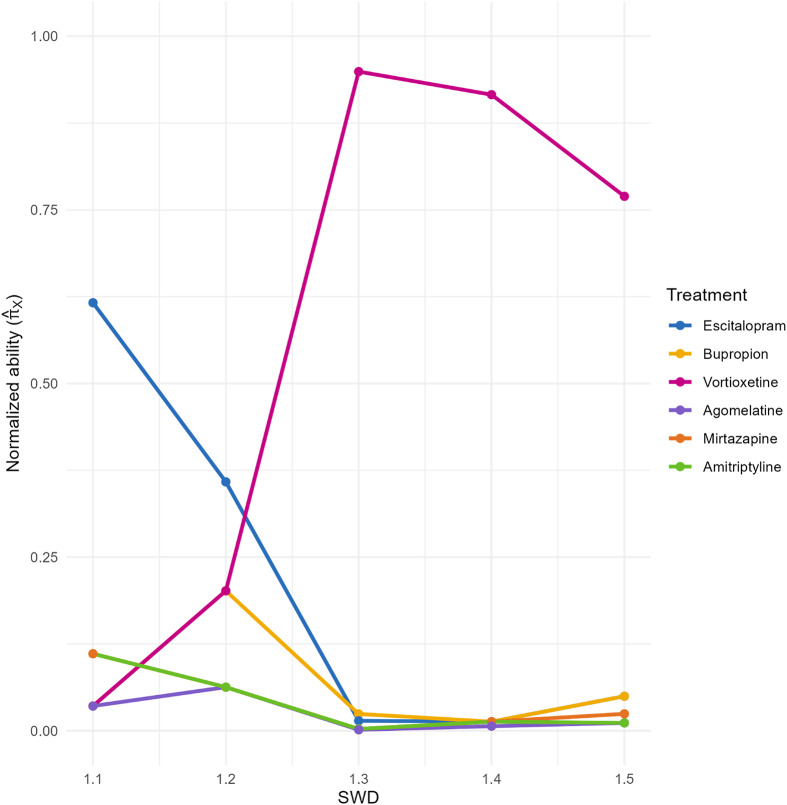
Sensitivity analysis for the network of antidepressants. The *y*-axis represents the probability of each treatment having the highest true ability and the *x*-axis the different SWD values. Figure 4 Long description.

Setting again an SWD of 1.20, we obtain the respective ROE that ranges from 0.83 to 1.20. Then, we applied the TCC of Section 2.1 to transform the 153 NMA estimates into treatment preferences. A high prevalence of ties was observed in the network, as only 32% of all comparisons yielded clinically important NMA estimates according to the defined TCC. The log-ability estimates (i.e., $\log\left({\hat{\psi }}_{X}\right))$ are shown in Figure 3b, while the normalized ability estimates ${\hat{\pi }}_{X}$ are shown in Table 2. Overall, within the context of the predefined TCC, Escitalopram demonstrated the highest ability to fulfil the TCC and yield beneficial treatment effect estimates, followed by Vortioxetine and Bupropion which are tied at the second position. In addition to these three treatments, Amitriptyline, Mirtazapine, and Agomelatine were also found to have significantly greater abilities to yield clinically important effects than the average treatment in the network. Finally, we conducted a sensitivity analysis regarding the definition of the SWD, progressively increasing it by 0.10 increments from the value of 1.10 up to 1.50. The results are shown in Figure 4, where to improve visibility we presented the results only in terms of the normalized abilities ${\hat{\pi }}_{X}$ associated with the first six treatments, as per the primary analysis. This sensitivity analysis indicated that if smaller treatment effects are of interest (i.e., SWD ≤1.20), then Escitalopram outperforms the other treatments. However, as the SWD increases, meaning that larger treatment effects are of interest, Vortioxetine demonstrates a greater ability to yield clinically important treatment effect estimates compared to all other treatments in the network.

Overall, in this example, some disagreements were observed among the treatment hierarchies obtained from the different methods. In principle, none of these hierarchies is invalid. In such cases, meta-analysts should carefully consider their research question and choose the method that best aligns with it.^6^ The final decision on which method to use depends on whether clinical importance and uncertainty should be incorporated into the treatment ranking. If accounting for clinical importance is a priority, then either the proposed method or the P-scores (SWD) should be prioritized, as the other available metrics do not adjust their results accordingly. Conversely, if clinical importance is not a primary concern, any of the remaining ranking metrics could be used. The final choice should then depend on whether the uncertainty of the NMA estimates should be incorporated when producing the treatment hierarchy.

### 4.2 Antihypertensive treatments and the incident of diabetes

**Figure 5:**
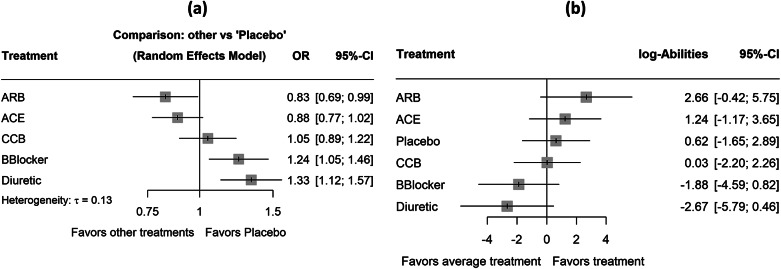
Forest plots with results for the network of antihypertensive treatments. (a) The summary odds ratios obtained assuming placebo as the reference treatment group. (b) The ranking results obtained using the proposed methodology. ACE, angiotensin-converting enzyme inhibitors; ARB, angiotensin receptor blockers; CCB, calcium channel blocker; BBlocker, beta blocker. Figure 5 Long description.

This network consists of 22 trials comparing five classes of antihypertensive treatments and placebo for the incidence of diabetes.^21^ This is a very well-connected network with 14 of the 15 possible direct comparisons being observed (Figure 2b). The primary outcome is the proportion of patients who developed diabetes and the NMA estimates using placebo as reference can be found in Figure 5a. The outcome is again measured as odds ratios (OR).

We consider again an SWD equal to 1.20^26^ and the respective ROE ranging from 0.83 to 1.20. The ranking results obtained from the approaches (a) to (d) can be found in Table 3, along with the NMA estimates of all treatments versus placebo, while the respective results in terms of median ranks are available in Table 2 in the Supplementary Material. In this network, small treatment effect values indicate beneficial effects. The results in terms of the estimated treatment abilities are depicted in Figure 5b, while the normalized ability estimates ${\hat{\pi }}_{X}$ are shown in Table 3.

**Table 3:** Ranking metrics for the network of antihypertensive drugs. Treatments with the top three values for each respective metric are shown in bold. The “Treatment” column is ordered according to P-scores Table 3 Long description.

Treatment	Odds ratio of treatment vs. placebo [position in ranking]	P-scores	P-scores (SWD)	PReTA	$p_{BV}$	${\hat{\pi }}_{X}$
ARB	**0.83 [1]**	**0.95**	**0.67**	**1.00**	**0.76**	**0.69**
ACE	**0.88 [2]**	**0.84**	**0.52**	**1.00**	**0.24**	**0.16**
Placebo	**1.00 [3]**	**0.55**	**0.32**	**0.77**	0.01	**0.09**
CCB	1.05 [4]	0.46	0.24	0.45	0.00	0.05
BBlocker	1.24 [5]	0.16	0.02	0.00	0.00	0.01
Diuretic	1.33 [6]	0.04	0.00	0.00	0.00	0.00

**Figure 6:**
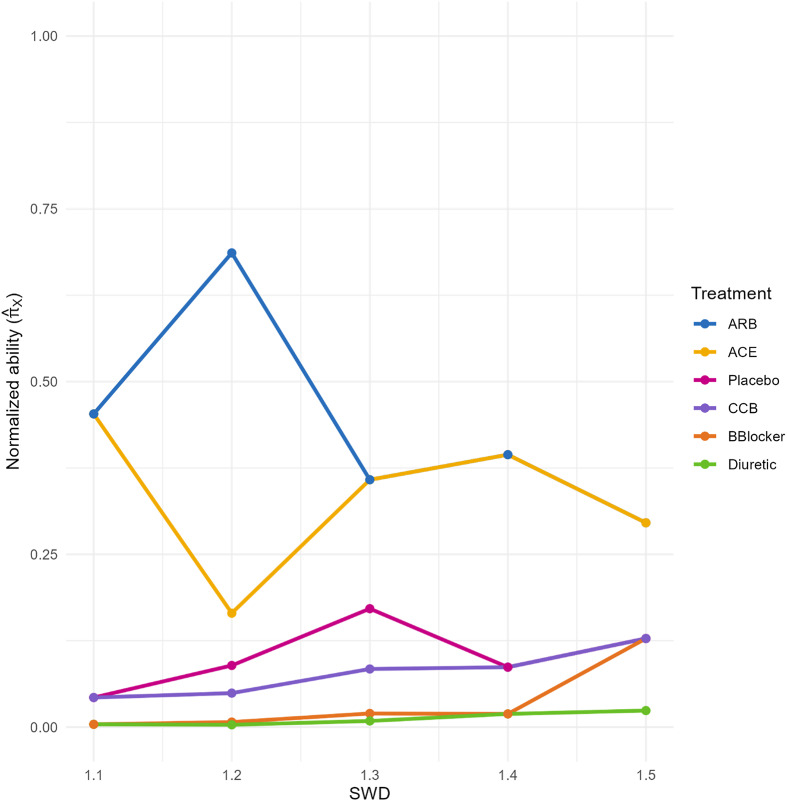
Sensitivity analysis for the network of the antihypertensive drugs. The y-axis represents the probability of each treatment having the highest true ability and the x-axis the different SWD values. Figure 6 Long description.

Based on the NMA estimates, ARB showed the most beneficial treatment effect, closely followed by ACE, which had a similar estimate in both magnitude and precision. Regarding the other ranking metrics, there is complete agreement across all five approaches, with ARB consistently ranked first. Notably, the TCC in this network indicated that 63% of all NMA estimates yielded a treatment preference. This perfect agreement among ranking methods can likely be attributed to the low uncertainty in the treatment effect estimates. Specifically, the standard errors of the NMA estimates range from 0.07 to 0.10 (Figure 3 in the Supplementary Material). Finally, to assess the robustness of the estimated rankings with respect to the definition of the TCC, we performed a sensitivity analysis, progressively increasing the SWD in 0.10 increments from the recommended value of 1.20 up to 1.50. The results are shown in Figure 6. Overall, this sensitivity analysis showed that ARB and ACE remained the top two treatments across the different SWD values.

## 5 Empirical investigation across 153 published networks

### 5.1 Database

We studied the agreement across different ranking metrics by reanalyzing networks from a database of published NMAs between 1999 and 2015, which included at least four treatments. To access these data, we used the R package nmadb.^44^ More details about this database can be found in the original publications.^22^^,^ ^23^ In this database, 267 datasets were identified with available data. Given that there was no information regarding the SWD across these 267 networks, we used the recommendations from previous publications, which suggested that a common choice for the SWD in the case of the risk ratio (RR) would be a value of 1.25.^27^^,^ ^45^ We therefore further restricted the database to include only networks with a binary outcome of interest. This yielded a database of 186 networks. After reanalyzing these 186 networks, we obtained results from 174 networks, as 12 networks from nmadb^44^ had incompatible data that did allow to fit a NMA model. Finally, applying the proposed ranking method to the set of 174 networks further restricted the networks with results to 153, as in the remaining 21 networks only ties were identified by the TCC. The NMA estimates and network geometries of these 21 networks are available in Figures 3–23 in the Supplementary Material.

### 5.2 Evaluated methods and performance metrics

We evaluated the agreement of the five methods presented in Section 4 in the context of a random-effects NMA model. This resulted in a total of 10 pairwise agreement comparisons between the different ranking metrics. Agreement was measured using Pearson’s correlation coefficient, indicating the agreement in the ranking values obtained by each of the different ranking metrics. In other words, we investigated whether larger values in one ranking metric also corresponded to larger values in the other ranking metrics. This approach slightly deviates from previous works,^5^^,^ ^7^ which studied agreement between different ranking methods by investigating the agreement in the treatment order of the ranking list. This was not straightforward in our case, as the five methods of interest present the final treatment order in different ways (i.e., allowing for tied positions or always yielding an explicit order). Finally, we further investigated how the precision of the NMA estimates, as a measure of the total amount of information in the network, impacts the agreement between the proposed ability-based metric and the other ranking metrics. To this end, following Chioccia et al.,^5^ we contrasted the correlation coefficients from each of the 153 networks with the following measures:

- the average variance across the (T2) NMA estimates $\hat{\theta }$ ,
- the relative range of variances, defined as $\frac{\max\left\{\mathrm{var}\left(\hat{\theta }\right)\right\}-\min\left\{\mathrm{var}\left(\hat{\theta }\right)\right\}}{\max\left\{\mathrm{var}\left(\hat{\theta }\right)\right\}}$ .

### 5.3 Results

The results regarding the median correlation and the interquartile range (IQR) of correlations across the 153 networks are presented in Table 4. Overall, the proposed ability-based ranking metric was found to be strongly correlated with most other ranking metrics, as the median correlation coefficient was typically above 0.90. A similarly high level of agreement was observed among most of the alternative ranking methods. It is worth noting that the strong agreement between the P-scores, P-scores (SWD), and PReTA metrics was expected, given that P-scores (SWD) and PReTA are essentially variations of the standard P-scores approach. Finally, the agreement between $p_{BV}$ and the proposed method was generally moderate. The latter also applies in terms of the agreement between $p_{BV}$ and the rest of the evaluated method, with the correlation becoming stronger primarily when $p_{BV}$ was compared to the P-scores adjusted for SWD.

**Table 4:** Pairwise agreement between the different ranking metrics, measured by the median Pearson’s correlation coefficient and the interquartile range of values obtained across 153 published NMAs Table 4 Long description.

	Median correlation [IQR]
log-Abilities vs. P-scores	0.95 [0.91, 0.97]
log-Abilities vs. $p_{BV}$	0.65 [0.52, 0.80]
log-Abilities vs. PReTA	0.93 [0.87, 0.96]
log-Abilities vs. P-scores (SWD)	0.93 [0.88, 0.97]
P-scores vs. $p_{BV}$	0.78 [0.71, 0.85]
P-scores vs. PReTA	0.97 [0.96, 0.98]
P-scores vs. P-scores (SWD)	0.99 [0.98, 1.00]
$p_{BV}$ vs. PReTA	0.70 [0.59, 0.81]
$p_{BV}$ vs. P-scores (SWD)	0.86 [0.77, 0.90]
PReTA vs. P-scores (SWD)	0.96 [0.94, 0.98]

**Figure 7:**
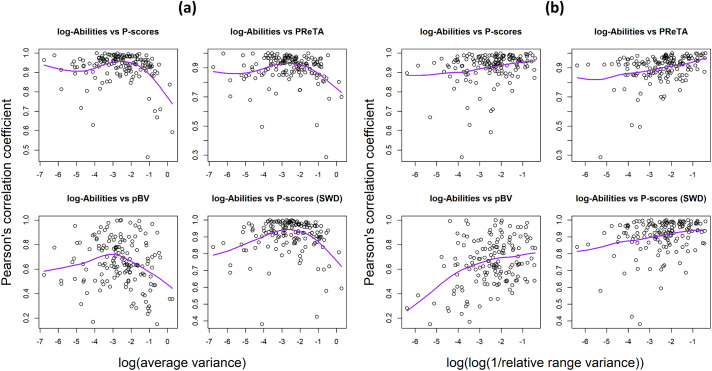
Scatter plots contrasting the correlation between the ability-based metric and the other ranking metrics across 153 networks. (a) The correlations plotted against the average variance of the NMA estimates; values on the left-hand side of the graph indicate greater precision. (b) The correlations plotted against the relative range of variances of the NMA estimates; values on the left-hand side of the graph indicate a larger variance range. In all scatter plots, the purple line represents a cubic smoothing spline with five degrees of freedom. Figure 7 Long description.

Figure 7 shows the results regarding the impact of uncertainty in the NMA estimates on the agreement between the ability-based metric and the other ranking metrics. Overall, the results indicate that this agreement depends on the level of uncertainty in the NMA estimates, with greater agreement observed in networks where estimates have higher precision and similar levels of uncertainty. In panel (a), the different correlation coefficients were plotted against the average variance of the NMA estimates, which were log-transformed to enhance visibility. The overall trend suggests that as the average variance of the NMA estimates increases, the correlation between the ability-based metric and the other ranking metrics decreases. In panel (b), the correlation coefficients were plotted against the relative range of variances. Following previous studies,^5^ the *x*-axis values were transformed using the double logarithm of the inverse relative range, so that values on the left-hand side indicate a larger variance range. These results showed that as the range of variances across the NMA estimates decreases, the agreement between the ability-based metric and the other ranking approaches increases. In other words, greater agreement is achieved in networks where the NMA treatment effects are estimated with similar levels of uncertainty. This is in line with previous empirical results that evaluated the rest of the approaches in terms of the same metrics.^5^^,^ ^7^

## 6 Discussion

In this article, we introduce a novel framework for producing treatment hierarchies in NMA through a probabilistic ranking model that accounts for a predefined TCC. The rationale behind the proposed ranking method differs from existing approaches, as it combines the NMA estimates with a concrete TCC or, in other words, a decision rule into a treatment hierarchy, whereas existing methods translate NMA estimates directly into rankings.

Our approach follows the principles of a typical decision-making process where a concrete decision rule is applied to the available evidence to translate the numerical results into practice.^24^^,^ ^28^^,^ ^29^^,^ ^46^^,^ ^47^ We start by applying the predefined TCC to the NMA relative treatment effects, transforming them into treatment preference data. We propose as a clinically relevant TCC the ROE between two treatments that represents the area within which their relative effect lacks indication of a treatment preference.^24^^,^ ^26^ Following previous work, we define the ROE using the SWD and its reciprocal (or opposite) value.^24^^,^ ^26^ Here, we propose a simple way for defining an ROE-based TCC based on the magnitude of the NMA treatment effect and its uncertainty. However, any TCC considered appropriate and clinically relevant can be used by investigators to produce preference data.

We parameterize our model to estimate the ability of each treatment to outperform the other treatments in the network^33^^,^ ^34^^,^ ^36^; that is, a latent characteristic referring to the propensity of each treatment in the network to yield clinically important and beneficial true treatment effects in the context of the defined TCC. Consequently, treatments with larger ability estimates corresponding to higher positions in the final ranking. Confidence intervals can also be placed next to the ability estimates to representing the uncertainty around the ranking metric. This should not be confused with other metrics proposed to evaluate the uncertainty of the treatment hierarchy.^48^ Furthermore, the interpretation of the ability estimates also stems from their transformation into probabilities using Luce’s axiom of choice.^37^^,^ ^38^ Model diagnostics were recently developed and can also be investigated in cases where no ties are identified from the TCC.^49^ However, these have not yet expanded to allow for ties. Overall, our method aims to produce clinically relevant treatment hierarchies accompanied by uncertainty measures. Of course, the proposed ranking method, like all existing ranking metrics, is not a substitute of the NMA relative effects; instead, it can be used to assist decision making and treatment recommendations.

Establishing a direct one-to-one mathematical relationship between the true ability of each treatment and the true treatment effects is challenging, as the former is a latent characteristic dependent on the TCC, while the latter is a fixed unknown parameter. This complicates the design of simulation studies, which typically begin by defining true treatment effects. However, this challenge is not unique to our method but applies broadly to treatment ranking in NMA, as the scope of the existing ranking metrics is to summarize evidence based on NMA estimates and they cannot be calculated directly from true treatment effect values.

We used two published networks to assess the properties of our method and compare it with existing approaches. Τhe network of antidepressants^20^ represents an extreme case as the treatment ranked highest in terms of effect size (Vortioxetine) yielded the least precise NMA estimates. Using a TCC defined according to the SWD reported in the original publication,^20^ our method produced more conservative results than the other methods, particularly regarding Vortioxetine’s position in the ranking. In a sensitivity analysis where we progressively increased the SWD, Vortioxetine moved to the top of the treatment hierarchy, reflecting its larger NMA estimate relative to other treatments. In the second network of antihypertensive treatments,^21^ we found a perfect agreement in the final ranking across all approaches. This agreement can be partly attributed to the high precision and narrow variance range of the NMA estimates.

We further explored the performance of the proposed framework and other common ranking metrics through a reanalysis of 153 published networks obtained from a published database,^22^^,^ ^23^ accessed via the R package nmadb.^44^ This empirical study showed strong agreement among most of the evaluated ranking metrics, except for $p_{BV}$ , which exhibited only moderate agreement with the others. We also investigated how the total amount of information in a network, expressed as the uncertainty in NMA estimates, affects the agreement between the proposed ability-based metric and the other methods. The results indicated that agreement depends on the level of uncertainty: greater agreement was observed in networks where NMA estimates had higher precision and similar levels of uncertainty across treatments.

We see several advantages of our proposed treatment ranking approach. First, the requirement of a priori defining a concrete TCC enables researchers to consider early on what constitutes a preferred treatment. In our approach, we estimate the treatment ability using maximum likelihood theory, thereby allowing us to obtain the standard error of the estimated abilities and infer about the uncertainty of ranking positions using standard statistical measures. In addition, the proposed model does not provide treatment ability estimates when all the NMA treatment effect estimates indicate ties due to convergence failure. Although this might be considered as a drawback of the model, we see it also as a way of preventing researchers from making ranking statements in the absence of sufficient evidence that the NMA estimates fulfil the TCC. This is in line with previous NMA recommendations for avoiding the presentation of ranking results in the presence of large uncertainty in the relative effects.^5^

Overall, in the presence of unstable conditions (e.g., sparse networks, rare events, etc.) NMA estimates are often biased and imprecise,^50^^–^ ^52^ thereby undermining the validity of any ranking method. When estimates are highly uncertain, the TCC defined in Section 2.1 is likely to fail to identify clinically meaningful effects, serving as a safeguard against presenting treatment hierarchies based on weak or unreliable evidence. This property was demonstrated in Section 5, where the TCC identified only ties across 21 sparse networks. Ultimately, the validity of any ranking method depends critically on the robustness of the underlying NMA estimates; hence, when the estimation of treatment effects is unstable, we recommend that researchers should be particularly cautious when in presenting and interpreting ranking results.

Despite these advantages, our approach is not free of limitations. Probably the most important limitation relates to the definition of the SWD and of the respective ROE that involves some subjectivity.^30^ On the other hand, though, the use of different ROEs allows researchers to estimate the treatment hierarchy under different settings (e.g., for different patient profiles). Ways to mitigate this inherent subjectivity have been suggested in the literature through fully statistical approaches^25^ or by incorporating information from patients.^25^ Moreover, investigators conducting NMAs may choose to define another TCC not based on the ROE. To avoid data-driven decisions, we recommend meta-analysts using our ranking method to define and justify the TCC they plan to use in their protocol and investigate the robustness of the estimated hierarchy under different SWD values.

Our proposed framework offers a novel alternative to existing ranking metrics for estimating treatment hierarchies in NMA. The importance of a well-defined treatment hierarchy question prior to estimating treatment ranking has been highlighted recently.^6^ To our knowledge, this is the first approach that incorporates explicitly and quantitatively considerations on the treatment hierarchy question through the predefined TCC. Future extensions of the proposed approach could include adapting the model to account for treatment-level characteristics (e.g., treatment cost) and multiple outcomes. The former is currently possible only in cases where no ties are allowed from the TCC.^54^ Overall, investigators can use the proposed approach either as their primary ranking tool or as sensitivity analysis alongside conventional ranking metrics particularly for networks with increased uncertainty in their relative effects and knowledge of clinically relevant TCC.

## Supporting information

10.1017/rsm.2026.10071.sm001 Evrenoglou et al. supplementary material Evrenoglou et al. supplementary material

## Data Availability

The full code and data to reproduce the results of the two illustrative examples and the empirical study are freely available on Zenodo using the following link: https://doi.org/10.5281/zenodo.18171269.

## Supplementary material

To view supplementary material for this article, please visit http://doi.org/10.1017/rsm.2026.10071.

## Long descriptions

### Figure 1 Long description

The left panel is a forest plot titled N M A treatment effect estimates for the comparison of 8 treatments versus a common reference Y, Type of outcome: Beneficial. The vertical axis lists comparisons from A versus Y down to H versus Y.

Data points are represented by squares with horizontal error bars.

- A versus Y, B versus Y, and C versus Y are red, located to the left of the null value.

- D versus Y, E versus Y, and F versus Y are black, centered near the null value.

- G versus Y and H versus Y are red, located to the right of the null value.

The horizontal axis features a solid vertical line at the null value. Two dashed vertical lines represent L super R O E to the left and U super R O E to the right. Below the axis, an arrow pointing left is labeled Favors Y, and an arrow pointing right is labeled Favors other treatments. A legend at the bottom left indicates red squares with bars represent Treatment preference and black squares with bars represent Tie.

The right panel is titled Output after applying T C C with a sub-header Treatment-choice. It lists the categorical outcomes for each comparison:

- Y is greater than A

- Y is greater than B

- Y is greater than C

- D equals Y

- E equals Y

- F equals Y

- G is greater than Y

- H is greater than Y

Navigate back to Figure 1.

### Table 1 Long description

The table consists of five columns and six rows.

Column headers from left to right are:

1. Characteristic (unlabeled in header)

2. Proposed ability-based metric (psi-hat sub X)

3. P-scores (or P R e T A)

4. p sub B V

5. P-scores (S W D)

Row 1: What is the treatment hierarchy question?

- Proposed metric: Based on predefined T C C, what is the propensity of treatment X to yield beneficial true effects compared to others?

- P-scores: Proportion of treatments inferior to X.

- p sub B V: Probability that each treatment yields the largest true effect.

- P-scores (S W D): Proportion of treatments inferior to X by a margin at least equal to S W D.

Row 2: How results are interpreted?

- Proposed metric: Ability estimate measures propensity to fulfill T C C; relative comparison is key.

- P-scores: Proportion of treatments worse than X.

- p sub B V: Probability that X yields the largest true effect.

- P-scores (S W D): Proportion of treatments worse than X by a margin of S W D.

Row 3: Does it account for clinical importance?

- Proposed metric: Yes, via T C C and Bradley-Terry model.

- P-scores: No.

- p sub B V: No.

- P-scores (S W D): Yes, relies on probability that N M A estimates are larger than S W D.

Row 4: Impact of clinical importance in the hierarchy

- Proposed metric: Hierarchy can change across different S W D values.

- P-scores: Not applicable.

- p sub B V: Not applicable.

- P-scores (S W D): Hierarchy does not change; always yields the same hierarchy as standard P-scores.

Row 5: Availability of ranking

- Proposed metric: Not available if only ties are identified by T C C.

- P-scores: Always available.

- p sub B V: Always available.

- P-scores (S W D): Always available.

Navigate back to Table 1.

### Figure 2 Long description

The first panel labeled a shows a dense network of eighteen teal circular nodes arranged in a large circle. Starting from the top and moving clockwise, the nodes are agomelatine, amitriptyline, bupropion, citalopram, clomipramine, duloxetine, escitalopram, fluoxetine, fluvoxamine, milnacipran, mirtazapine, nefazodone, paroxetine, reboxetine, sertraline, trazodone, venlafaxine, and vortioxetine. Numerous gray lines of varying thickness connect the nodes. Notably thick lines connect amitriptyline to fluoxetine and paroxetine, and fluoxetine to sertraline.

The second panel labeled b shows a hexagonal network of six teal circular nodes. Starting from the top and moving clockwise, the nodes are A C E, A R B, BBlocker, C C B, Diuretic, and Placebo. Lines connect every node to every other node. The thickest lines connect A C E to C C B, A C E to Diuretic, and C C B to BBlocker. Thinner lines connect the remaining pairs such as Placebo to A R B and A R B to BBlocker.

Navigate back to Figure 2.

### Table 2 Long description

The table consists of seven columns: Treatment, Odds ratio of treatment vs. placebo [position in ranking], P-scores, P-scores (S W D), P R e T A, and two columns with mathematical symbols for pi-sub-B V and wide-hat-pi-sub-X.

Top ranked treatments include:

* Vortioxetine: Odds ratio 1.87 [1], P-score 0.90, P-score (S W D) 0.75, P R e T A 0.93, pi-sub-B V 0.64, wide-hat-pi-sub-X 0.20.

* Escitalopram: Odds ratio 1.51 [3], P-score 0.83, P-score (S W D) 0.49, P R e T A 0.98, pi-sub-B V 0.07, wide-hat-pi-sub-X 0.36.

* Bupropion: Odds ratio 1.55 [2], P-score 0.79, P-score (S W D) 0.53, P R e T A 0.87, pi-sub-B V 0.19, wide-hat-pi-sub-X 0.20.

Other treatments listed in descending order of P-score: Mirtazapine (0.75), Amitriptyline (0.71), Agomelatine (0.64), Paroxetine (0.62), Venlafaxine (0.61), Duloxetine (0.52), Milnacipran (0.49), Sertraline (0.45), Nefazodone (0.38), Citalopram (0.37), Clomipramine (0.26), Fluvoxamine (0.25), Fluoxetine (0.23), Trazodone (0.12), and Reboxetine (0.09).

Values for Odds Ratio generally decrease with the P-score ranking, with Reboxetine showing the lowest performance at 0.95 [18].

Navigate back to Table 2.

### Figure 3 Long description

Two forest plots labeled a and b.

Panel a is titled Comparison other versus Trazodone Random Effects Model. The x-axis at the bottom is logarithmic, centered at 1, with labels 0.5, 1, and 2. Values below 1 favor Trazodone and values above 1 favor other treatments. The list of 17 treatments from top to bottom includes Vortioxetine, Bupropion, Escitalopram, Mirtazapine, Amitriptyline, Agomelatine, Paroxetine, Venlafaxine, Duloxetine, Milnacipran, Sertraline, Citalopram, Nefazodone, Fluoxetine, Clomipramine, Fluvoxamine, and Reboxetine. Vortioxetine has the highest O R of 1.87 with a 95 percent C I of 1.04 to 3.38. Reboxetine has the lowest O R of 0.95 with a 95 percent C I of 0.64 to 1.41. Heterogeneity tau equals 0.18.

Panel b displays ranking results using log-Abilities. The x-axis ranges from negative 5 to 5, centered at 0. Values below 0 favor average treatment and values above 0 favor treatment. The treatments are ranked from highest to lowest log-Abilities. Escitalopram is at the top with a value of 4.49 and a 95 percent C I of 2.12 to 6.86. The list continues through Vortioxetine, Bupropion, Amitriptyline, Mirtazapine, Agomelatine, Duloxetine, Paroxetine, Milnacipran, Venlafaxine, Nefazodone, Sertraline, Citalopram, Fluvoxamine, Clomipramine, and Fluoxetine. Reboxetine and Trazodone are at the bottom, both with a log-Ability of negative 6.52 and a 95 percent C I of negative 9.14 to negative 3.90.

Navigate back to Figure 3.

### Figure 4 Long description

The y-axis is labeled Normalized ability pi-hat sub x and ranges from 0.00 to 1.00. The x-axis is labeled S W D with five discrete points at 1.1, 1.2, 1.3, 1.4, and 1.5.

Six treatments are plotted as colored lines with circular data points.

* Vortioxetine (magenta) starts low at 0.04, rises to 0.20 at 1.2, then sharply peaks at 0.95 at 1.3, remaining high at 0.91 at 1.4 and 0.77 at 1.5.

* Escitalopram (blue) starts at its highest point of 0.62 at 1.1, drops to 0.36 at 1.2, and then falls to near zero for the remaining values.

* Bupropion (yellow) starts near zero, shows a slight peak of 0.03 at 1.3, and ends at 0.05 at 1.5.

* Agomelatine (purple) starts at 0.04, peaks slightly at 0.07 at 1.2, and then drops to zero at 1.3.

* Mirtazapine (orange) starts at 0.11 and trends downward to near zero by 1.3, with a slight rise to 0.03 at 1.5.

* Amitriptyline (green) starts at 0.11 and trends downward to zero by 1.3, remaining at zero through 1.5.

Navigate back to Figure 4.

### Figure 5 Long description

Two-panel forest plot.

Panel A is titled Comparison other vs Placebo Random Effects Model. The x-axis ranges from 0.75 to 1.5 with a vertical line at 1. Values less than 1 favor other treatments and values greater than 1 favor Placebo. Heterogeneity tau equals 0.13.

Data points from top to bottom:

- A R B: O R 0.83, 95 percent C I 0.69 to 0.99.

- A C E: O R 0.88, 95 percent C I 0.77 to 1.02.

- C C B: O R 1.05, 95 percent C I 0.89 to 1.22.

- BBlocker: O R 1.24, 95 percent C I 1.05 to 1.46.

- Diuretic: O R 1.33, 95 percent C I 1.12 to 1.57.

Panel B shows ranking results. The x-axis ranges from negative 4 to 4 with a vertical line at 0. Values less than 0 favor average treatment and values greater than 0 favor treatment.

Data points from top to bottom:

- A R B: log-Abilities 2.66, 95 percent C I negative 0.42 to 5.75.

- A C E: log-Abilities 1.24, 95 percent C I negative 1.17 to 3.65.

- Placebo: log-Abilities 0.62, 95 percent C I negative 1.65 to 2.89.

- C C B: log-Abilities 0.03, 95 percent C I negative 2.20 to 2.26.

- BBlocker: log-Abilities negative 1.88, 95 percent C I negative 4.59 to 0.82.

- Diuretic: log-Abilities negative 2.67, 95 percent C I negative 5.79 to 0.46.

Navigate back to Figure 5.

### Table 3 Long description

The table consists of seven columns and seven rows including the header. The columns are: Treatment, Odds ratio of treatment versus placebo [position in ranking], P-scores, P-scores (S W D), P R e T A, absolute value of B V, and pi-hat sub X.

* Row 1 (A R B): Odds ratio 0.83 [1], P-score 0.95, P-score (S W D) 0.67, P R e T A 1.00, absolute value of B V 0.76, pi-hat sub X 0.69.

* Row 2 (A C E): Odds ratio 0.88 [2], P-score 0.84, P-score (S W D) 0.52, P R e T A 1.00, absolute value of B V 0.24, pi-hat sub X 0.16.

* Row 3 (Placebo): Odds ratio 1.00 [3], P-score 0.55, P-score (S W D) 0.32, P R e T A 0.77, absolute value of B V 0.01, pi-hat sub X 0.09.

* Row 4 (C C B): Odds ratio 1.05 [4], P-score 0.46, P-score (S W D) 0.24, P R e T A 0.45, absolute value of B V 0.00, pi-hat sub X 0.05.

* Row 5 (B Blocker): Odds ratio 1.24 [5], P-score 0.16, P-score (S W D) 0.02, P R e T A 0.00, absolute value of B V 0.00, pi-hat sub X 0.01.

* Row 6 (Diuretic): Odds ratio 1.33 [6], P-score 0.04, P-score (S W D) 0.00, P R e T A 0.00, absolute value of B V 0.00, pi-hat sub X 0.00.

Values for A R B and A C E are consistently the highest across most metrics.

Navigate back to Table 3.

### Figure 6 Long description

The vertical y-axis is labeled Normalized ability pi-hat sub x and ranges from 0.00 to 1.00. The horizontal x-axis is labeled S W D with discrete values at 1.1, 1.2, 1.3, 1.4, and 1.5.

Six treatments are plotted as colored lines with circular markers:

* A R B (blue line): Starts at 0.45, peaks sharply at 0.70 when S W D is 1.2, then drops to 0.38 at 1.3 and is not plotted further.

* A C E (yellow line): Starts at 0.45, drops to 0.18 at 1.2, rises to 0.38 at 1.3 and 1.4, then ends at 0.30.

* Placebo (magenta line): Shows a gradual rise from 0.05 to a peak of 0.18 at 1.3, then fluctuates down to 0.10 and back to 0.12.

* C C B (purple line): Maintains a low, steady increase from 0.05 at 1.1 to 0.12 at 1.5.

* B Blocker (orange line): Remains near the baseline, starting at 0.00 and ending at 0.02.

* Diuretic (green line): Remains at the baseline, starting at 0.00 and ending at 0.02.

Navigate back to Figure 6.

### Table 4 Long description

The table consists of two columns. The first column lists the metric pairs being compared, and the second column provides the Median correlation followed by the I Q R in brackets.

* log-Abilities versus P-scores: 0.95 [0.91, 0.97]

* log-Abilities versus p sub B V: 0.65 [0.52, 0.80]

* log-Abilities versus PReTA: 0.93 [0.87, 0.96]

* log-Abilities versus P-scores S W D: 0.93 [0.88, 0.97]

* P-scores versus p sub B V: 0.78 [0.71, 0.85]

* P-scores versus PReTA: 0.97 [0.96, 0.98]

* P-scores versus P-scores S W D: 0.99 [0.98, 1.00]

* p sub B V versus PReTA: 0.70 [0.59, 0.81]

* p sub B V versus P-scores S W D: 0.86 [0.77, 0.90]

* PReTA versus P-scores S W D: 0.96 [0.94, 0.98]

Navigate back to Table 4.

### Figure 7 Long description

The image consists of two main panels, a and b, each containing a two-by-two grid of scatter plots.

Panel a: The x-axis for all four plots is log open parenthesis average variance close parenthesis, ranging from negative 7 to 0. The y-axis is Pearson's correlation coefficient, ranging from 0.2 to 1.0.

- Top-left: log-Abilities versus P-scores. Data points are concentrated between 0.8 and 1.0 correlation. The purple spline curve peaks around x equals negative 3 and declines sharply toward x equals 0.

- Top-right: log-Abilities versus P R e T A. Similar distribution to P-scores but with slightly more vertical spread. The spline curve shows a gentle hump peaking at x equals negative 2.5.

- Bottom-left: log-Abilities versus p B V. Data is more dispersed, ranging from 0.2 to 1.0 correlation. The spline curve rises to a peak at x equals negative 3 and then declines.

- Bottom-right: log-Abilities versus P-scores open parenthesis S W D close parenthesis. High concentration of points near 1.0 correlation, with a spline curve that peaks at x equals negative 3.

Panel b: The x-axis for all four plots is log open parenthesis log open parenthesis 1 forward slash relative range variance close parenthesis close parenthesis, ranging from negative 6 to negative 1. The y-axis is Pearson's correlation coefficient.

- Top-left: log-Abilities versus P-scores. Points are clustered at high correlation values. The spline curve shows a slight upward trend as x increases.

- Top-right: log-Abilities versus P R e T A. Similar clustering to the P-scores plot with a slight upward-sloping spline.

- Bottom-left: log-Abilities versus p B V. Shows a distinct positive linear-like trend. The spline curve starts low at correlation 0.3 when x is negative 6 and rises steadily to 0.7 at x equals negative 1.

- Bottom-right: log-Abilities versus P-scores open parenthesis S W D close parenthesis. Points are densely packed at the top of the graph. The spline curve shows a gradual upward slope from 0.8 to 0.95.

Navigate back to Figure 7.
