# Supplementary material for: Producing treatment hierarchies in network meta-analysis using probabilistic models and treatment-choice criteria
Source: Res Synth Methods. 2026 Feb 20;17(4):750–69. doi: 10.1017/rsm.2026.10071 (PMC13311357; doi:10.1017/rsm.2026.10071)
Supplement: Evrenoglou et al. supplementary material [file S1759287926100714sup001.docx]

# Supplementary Material

## **Mathematical representation of the treatment choice criterion introduced in Section 2.1 of the main manuscript**

We introduce the mathematical representation of our TCC for the case of a beneficial outcome where larger treatment effects indicate better treatment performance. Suppose that $I_{XY}^{(1)}$ and $I_{XY}^{(2)}$ are two indicator variables defined for each pairwise comparison $XY$ in the network with $X,Y=1,2,\ldots,T$, as

| and | $I_{XY}^{(1)}=\left\{ \begin{aligned} 1, \mathrm{if}\left( \hat{\theta}_{XY}>U^{\mathrm{ROE}} \right) \mathrm{and} \left( l_{XY}>L^{ROE} \right) \\ \\ 0,\mathrm{otherwise} \end{aligned} \right.$ | (1) |
| --- | --- | --- |
|  | $I_{XY}^{(2)}=\left\{ \begin{aligned} -1,\mathrm{if}\left( \hat{\theta}_{XY}<L^{\mathrm{ROE}} \right) \mathrm{and} \left( u_{XY}<U^{ROE} \right) \\ \\ 0, \mathrm{otherwise} \end{aligned} \right.$ | (2) |

where $L^{\mathrm{ROE}}$ and $U^{\mathrm{ROE}}$ are the lower and upper limits of the ROE respectively. In this way the indicator variable $I_{XY}^{(1)}$ becomes 1 when the necessary TCC conditions for the treatment $Y$ to be preferred over treatment $X$ are satisfied and 0 elsewhere. Similarly, the indicator variable $I_{XY}^{(2)}$ becomes -1 when the necessary TCC conditions for treatment $X$ to be preferred over the treatment $Y$ are satisfied and 0 elsewhere. Then, the TCC for each comparison $XY$ can be defined for a beneficial outcome based on the following conditions:

|  | $Y>X, if \sum_{k=1}^{2} I_{XY}^{\left( k \right)}=1$  $X>Y, if \sum_{k=1}^{2} I_{XY}^{\left( k \right)}=-1$  $Y=X, if \sum_{k=1}^{2} I_{XY}^{\left( k \right)}=0$ | (3a)  (3b)  (3c) |
| --- | --- | --- |

where $Y>X$ or $X>Y$ indicate a treatment preference and $Y=X$ indicates a ‘tie’, representing cases where the available evidence from the NMA estimate for the comparison $XY$ does not support any clear treatment preference, based on the TCC. In case of a harmful outcome, we need to reverse the signs of 1 and -1 in Equations (3a) and (3b). Investigators who prefer to use a different TCC can modify Equations (1) and (2) accordingly. After defining the TCC, all the $\binom{T}{2}$ NMA estimates are transformed into a treatment preference format using Equations (3a)-(3c) for each comparison $XY$ in the network.

## **Analytical proof that the probability of a tie between two treatments is maximized when the treatments have equal abilities**

**Proposition:** The probability of a tie between two treatments, $X$ and $Y$, as given by Equation (9) in the main manuscript, is maximized when the two treatments have equal abilities, $\psi_{X}=\psi_{Y}$.

**Proof:** We start the proof by simplifying the notation setting $x=\psi_{X}$ and $y=\psi_{Y}$. Supposing that $y\neq0$ and writing $x=ry$ the Equation (9) from the main manuscript can be re-expressed as,

$P\left( X=Y \right)=\frac{\sqrt{r}vy}{ry+y+\sqrt{r}vy}=\frac{\sqrt{r}v}{r+1+\sqrt{r}v}$, with $v>0$ and $r>0$,

showing that the probability of tie only depends on $v$ and on the ratio $r$. Assuming that $v$ is fixed, we define $f\left( r \right)=\frac{\sqrt{r}v}{r+1+\sqrt{r}v}$. Taking the first derivative of $f(r)$ we get,

$$f^{'}\left( r \right)=\frac{\frac{v}{2\sqrt{r}}\left( r+1+v\sqrt{r} \right)-v\sqrt{r}\left( 1+\frac{v}{2\sqrt{r}} \right)}{\left( r+1+v\sqrt{r} \right)^{2}}=\ldots=\frac{v(1-r)}{2\sqrt{r}\left( r+1+v\sqrt{r} \right)^{2}}$$

Given that $r>0$and $v>0,$we get that $f^{'}\left( r \right)=0\Leftrightarrow r=1$. Finally, taking the second derivative of $f(r)$ we get,

$$f^{''}\left( r \right)=\frac{-v\left[ 2\sqrt{r}\left( r+1+v\sqrt{r} \right)^{2} \right]-v(1-r)\left[ \frac{1}{\sqrt{r}}\left( r+1+v\sqrt{r} \right)^{2}+4\sqrt{r}\left( r+1+v\sqrt{r} \right)\left( 1+\frac{v}{2\sqrt{r}} \right) \right]}{\left( 2\sqrt{r}\left( r+1+v\sqrt{r} \right)^{2} \right)^{2}}$$

with $f^{''}\left( 1 \right)=-\frac{v}{2\left( 2+v \right)^{2}}<0$. Wraping up everything, we have that for $f\left( r \right)=\frac{\sqrt{r}v}{r+1+\sqrt{r}v}$, $f^{'}\left( r \right)=0\Leftrightarrow r=1$ and $f^{''}\left( 1 \right)<0.$Therefore, the function $f(r)$ is maximized for $r=1$, or in other words the probability of a tie is maximized when $\psi_{X}= \psi_{Y}$.

**Additional results for the network of antidepressants**

**Table 1**: Median ranks alongside with a 95% confidence interval for the network of antidepressants.

| **Treatment** | **Median rank** | **95% CI** |
| --- | --- | --- |
| Vortioxetine | 1 | [1, 15] |
| Escitalopram | 3 | [1, 10] |
| Bupropion | 3 | [1, 15] |
| Mirtazapine | 5 | [1, 12] |
| Amitriptyline | 6 | [2, 12] |
| Agomelatine | 7 | [2, 15] |
| Paroxetine | 7 | [3, 12] |
| Venlafaxine | 7 | [3, 12] |
| Duloxetine | 9 | [2, 16] |
| Milnacipran | 10 | [2, 17] |
| Sertraline | 10 | [4, 15] |
| Nefazodone | 13 | [2, 18] |
| Citalopram | 12 | [5, 17] |
| Clomipramine | 14 | [6, 18] |
| Fluvoxamine | 14 | [6, 18] |
| Fluoxetine | 14 | [10, 17] |
| Trazodone | 17 | [10, 18] |
| Reboxetine | 17 | [10, 18] |


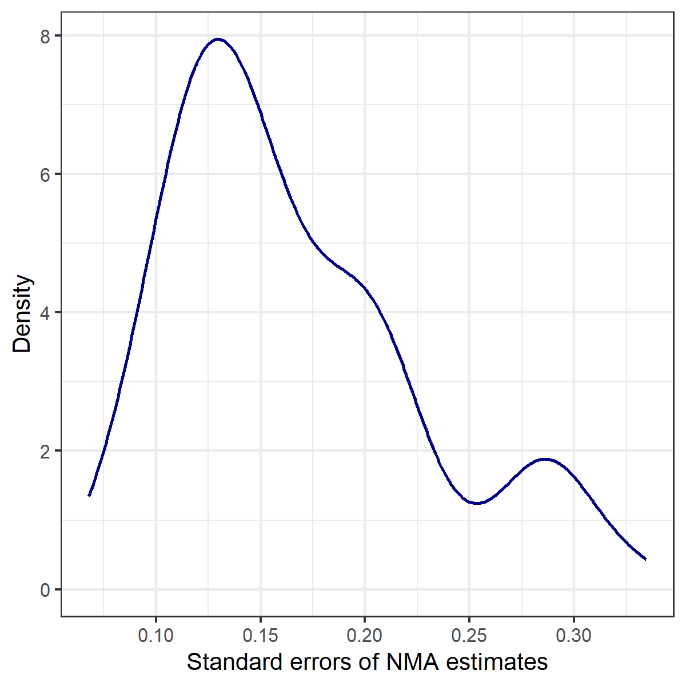


**Figure 1:** Standard error distribution across all 153 NMA estimates in the network of antidepressants.

## **Additional results for the network of antihypertensive drugs**

**Table 2:** Median ranks alongside with a 95% confidence interval for the network of antihypertensive drugs.

| **Treatment** | **Median rank** | **95% CI** |
| --- | --- | --- |
| ARB | 1 | [1, 2] |
| ACE | 2 | [1, 3] |
| Placebo | 3 | [2, 4] |
| CCB | 4 | [3, 4] |
| BBlocker | 5 | [5, 6] |
| Diuretic | 6 | [5, 6] |


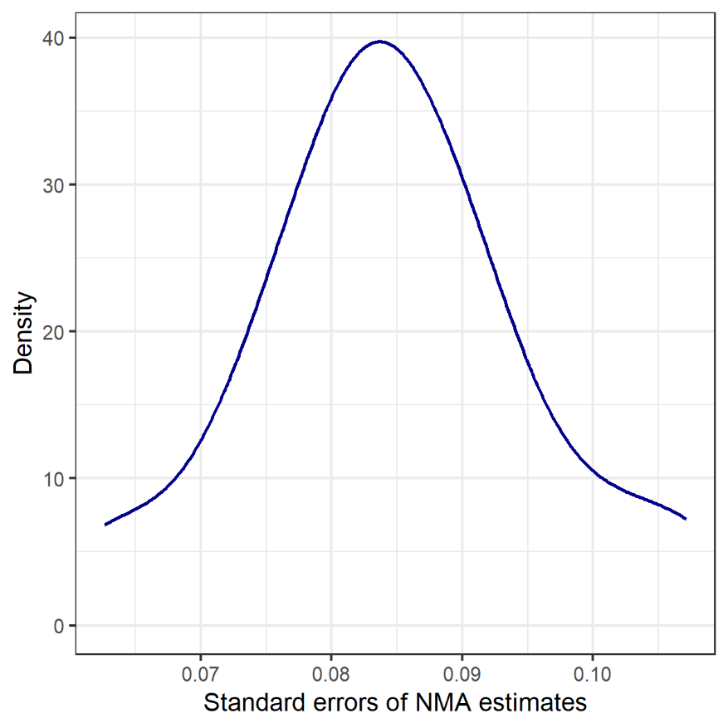


**Figure 2:** Standard error distribution across all 15 NMA estimates in the network of antihypertensive drugs.

## **Additional results from the empirical investigation across 153 published networks**

Among the 174 published networks that could potentially be re-analysed in the empirical study presented in the main manuscript, 21 were identified as containing only ties among their NMA estimates after applying the defined TCC in terms of a RR. As a result, the primary analysis was conducted using the proposed ability-based metric on the remaining 153 networks. In this section, we provide additional details about the 21 excluded networks, for which the proposed method could not be applied. The results for each of these networks, in terms of basic NMA estimates and rankings, are shown in panels (a) of **Figures 3–23** wilepanels (b) depict the network geometry of each of these networks.


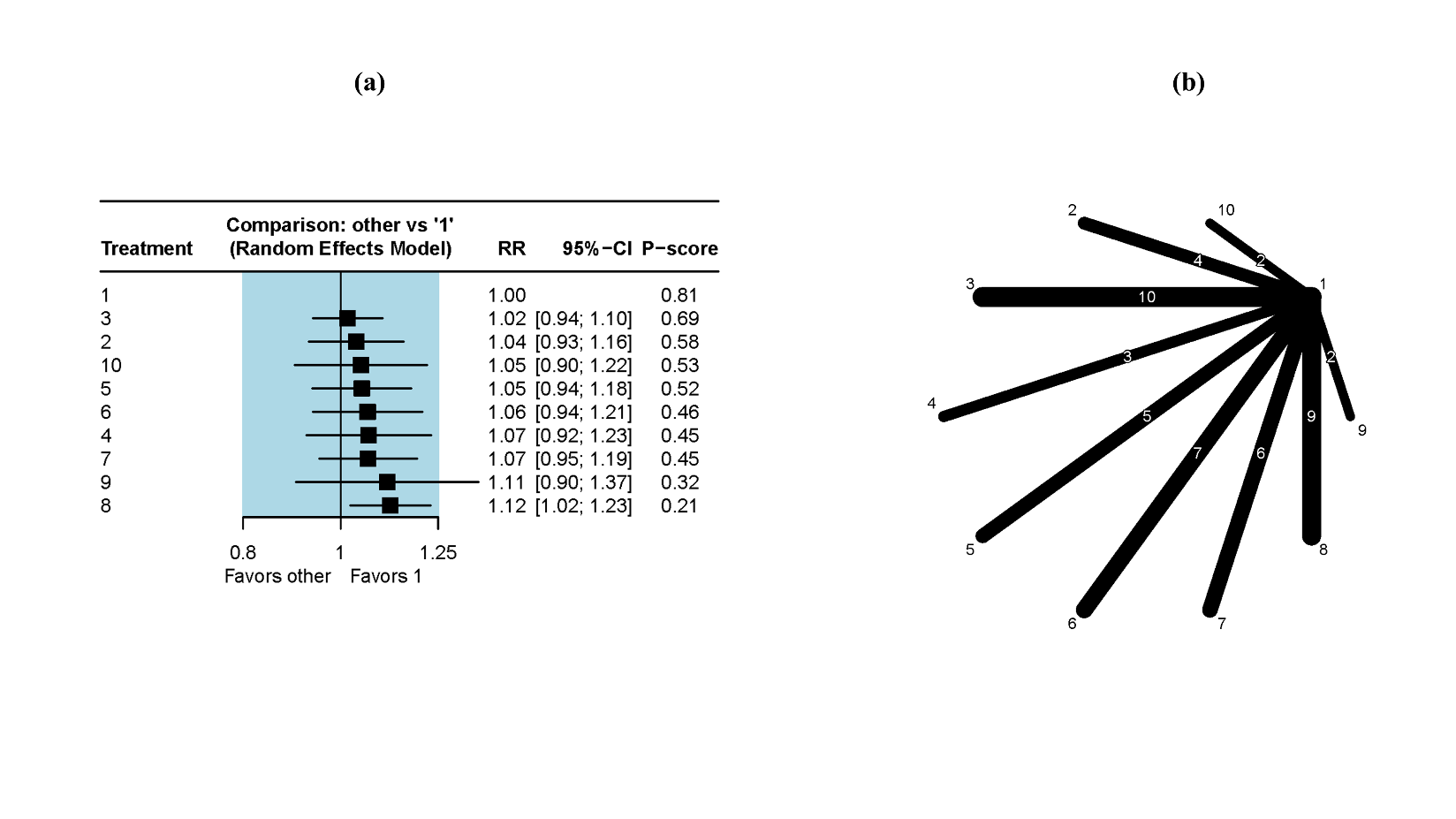
The median NMA estimate, on the natural scale, across the 21 networks was 1, with an interquartile range (IQR) of [0.93, 1.06]. In terms of outcome types, 13 networks had harmful outcomes, with a median NMA estimate and IQR of 1 [0.91, 1.05]. The remaining 8 networks had beneficial outcomes, with a median NMA estimate and IQR of 1 [0.94, 1.01]. Overall, across all networks, we observed small NMA estimates, close to the null effect. However, in several cases, the respective ranking results masked these small differences in NMA estimates and suggested a “clear order” in the ranking list (e.g., Figures 7–8 etc.). In such cases, interpreting the results based solely on ranking metrics could lead to an overinterpretation of the findings.

**Figure 3:** Results from the **first** of the 21 networks where only ties were identified. Panel (a) presents the NMA estimates and ranking in terms of P-scores, while Panel (b) shows the corresponding network graph. The blue area repreents the range of equivalence.


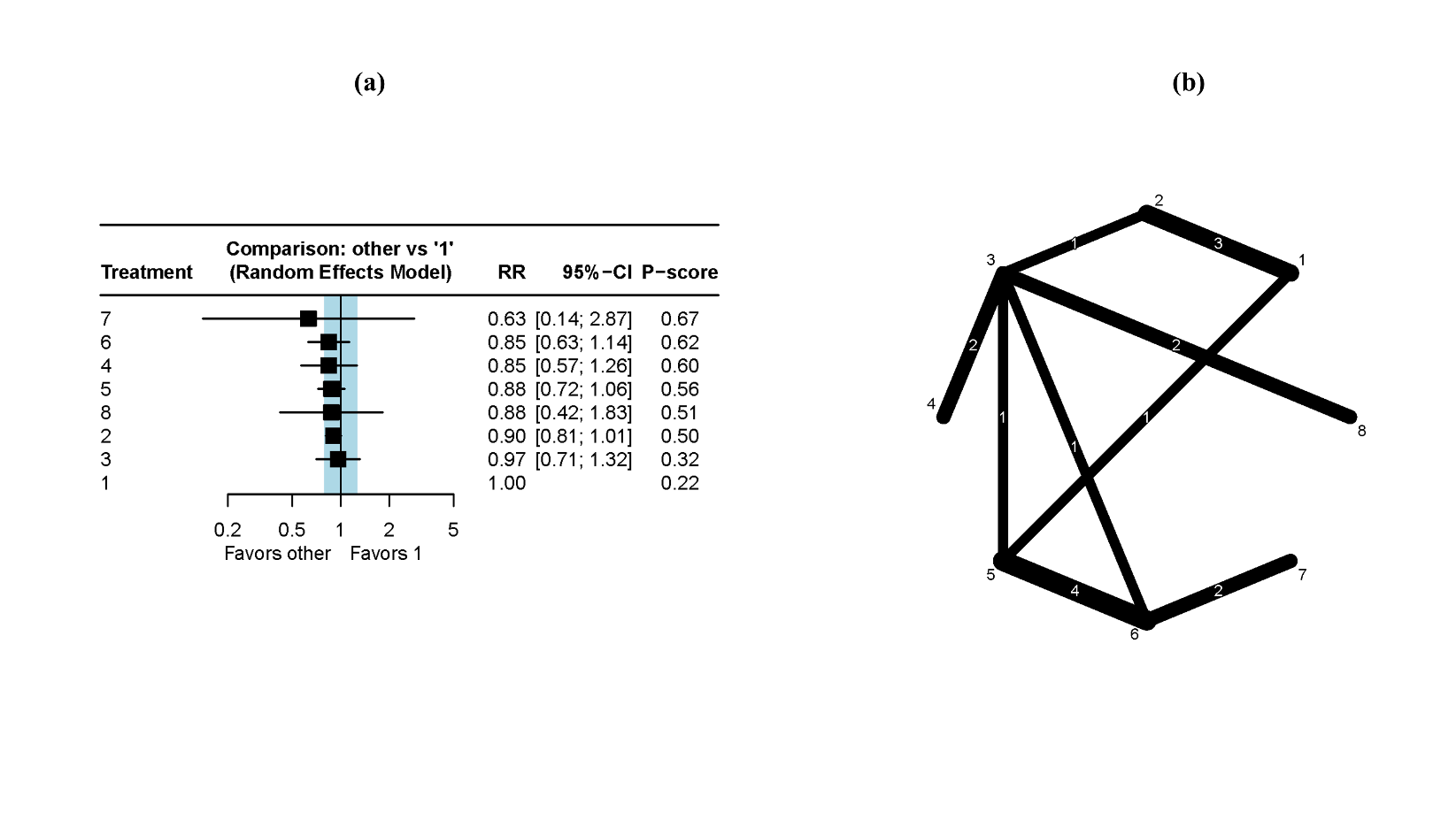

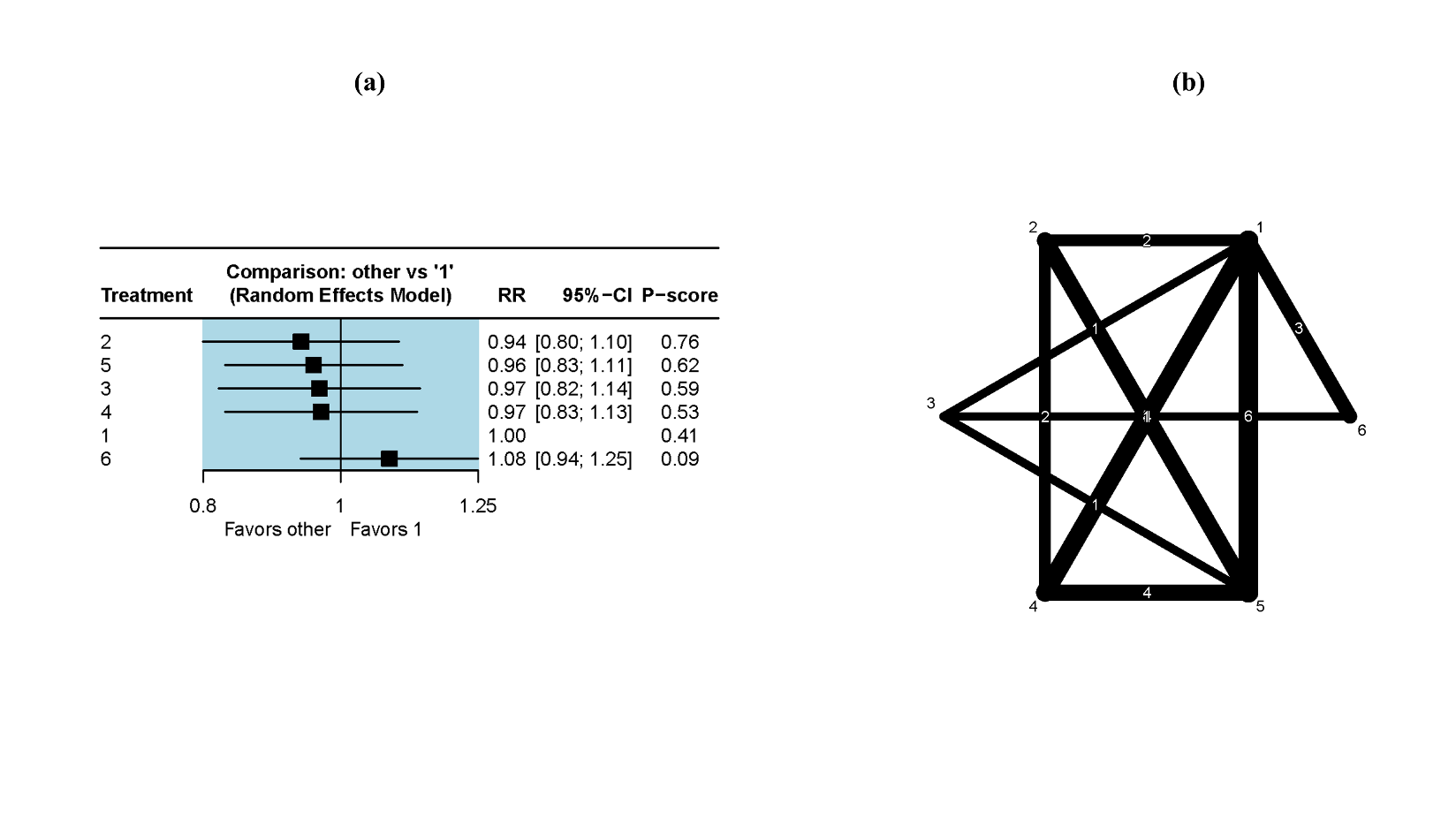


**Figure 5:** Results from the **third** of the 21 networks where only ties were identified. Panel (a) presents the NMA estimates and ranking in terms of P-scores, while Panel (b) shows the corresponding network graph. The blue area represents the range of equivalence.

**Figure 4**: Results from the **second** of the 21 networks where only ties were identified. Panel (a) presents the NMA estimates and ranking in terms of P-scores, while Panel (b) shows the corresponding network graph. The blue area represents the range of equivalence.


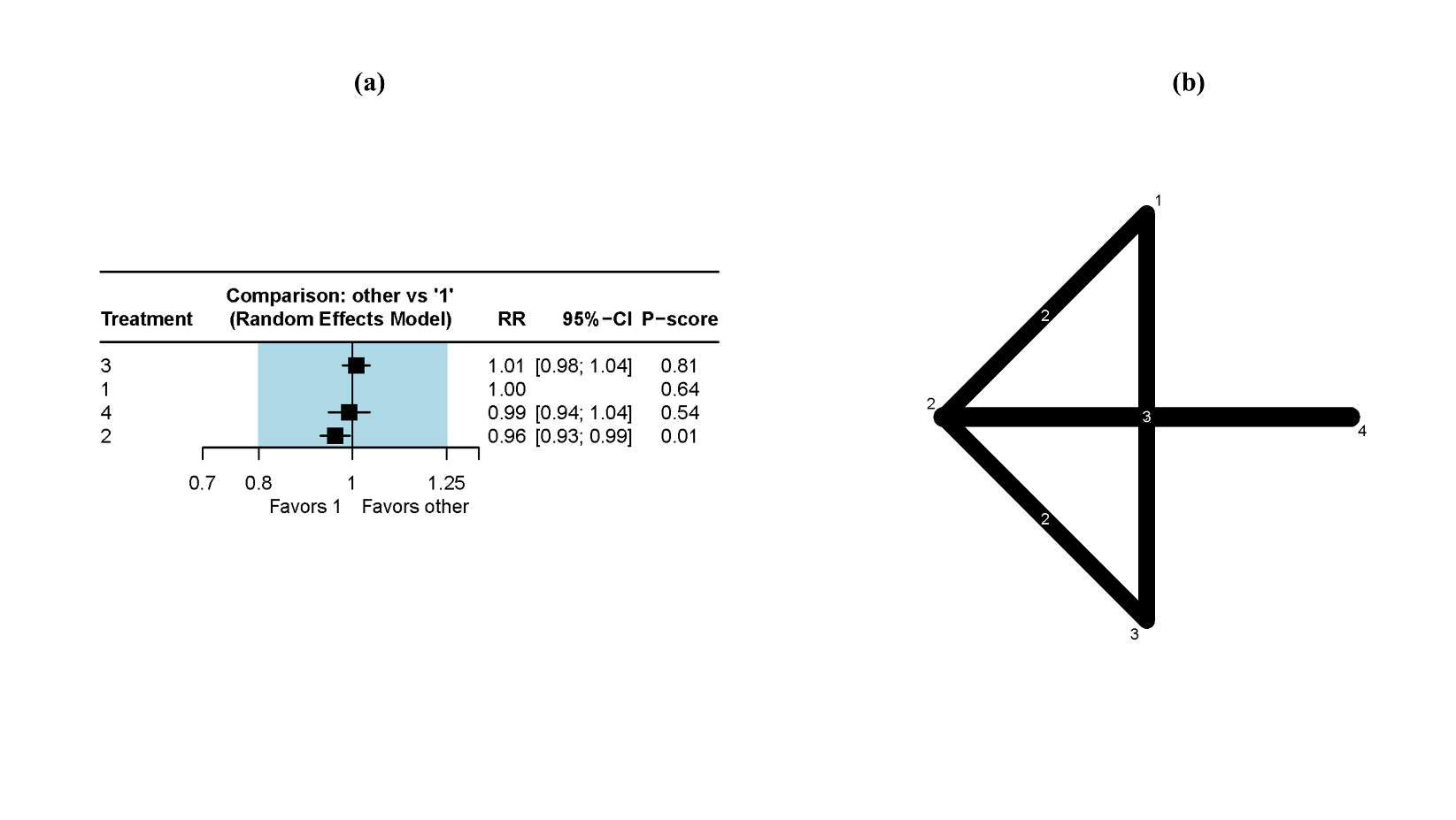

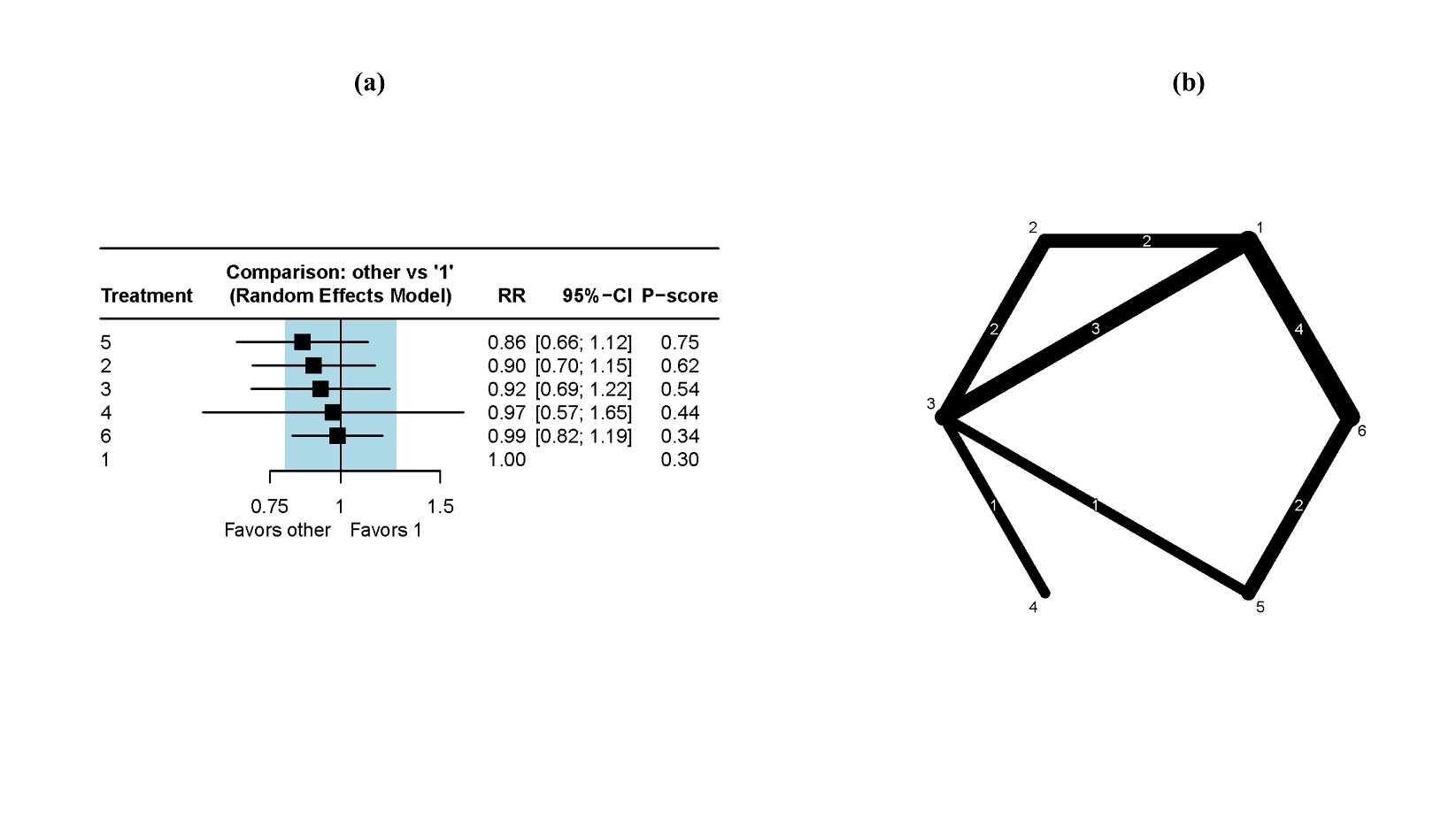


**Figure 7:** Results from the **fifth** of the 21 networks where only ties were identified. Panel (a) presents the NMA estimates and ranking in terms of P-scores, while Panel (b) shows the corresponding network graph. The blue area represents the range of equivalence.

**Figure 6:** Results from the **fourth** of the 21 networks where only ties were identified. Panel (a) presents the NMA estimates and ranking in terms of P-scores, while Panel (b) shows the corresponding network graph. The blue area represents the range of equivalence.


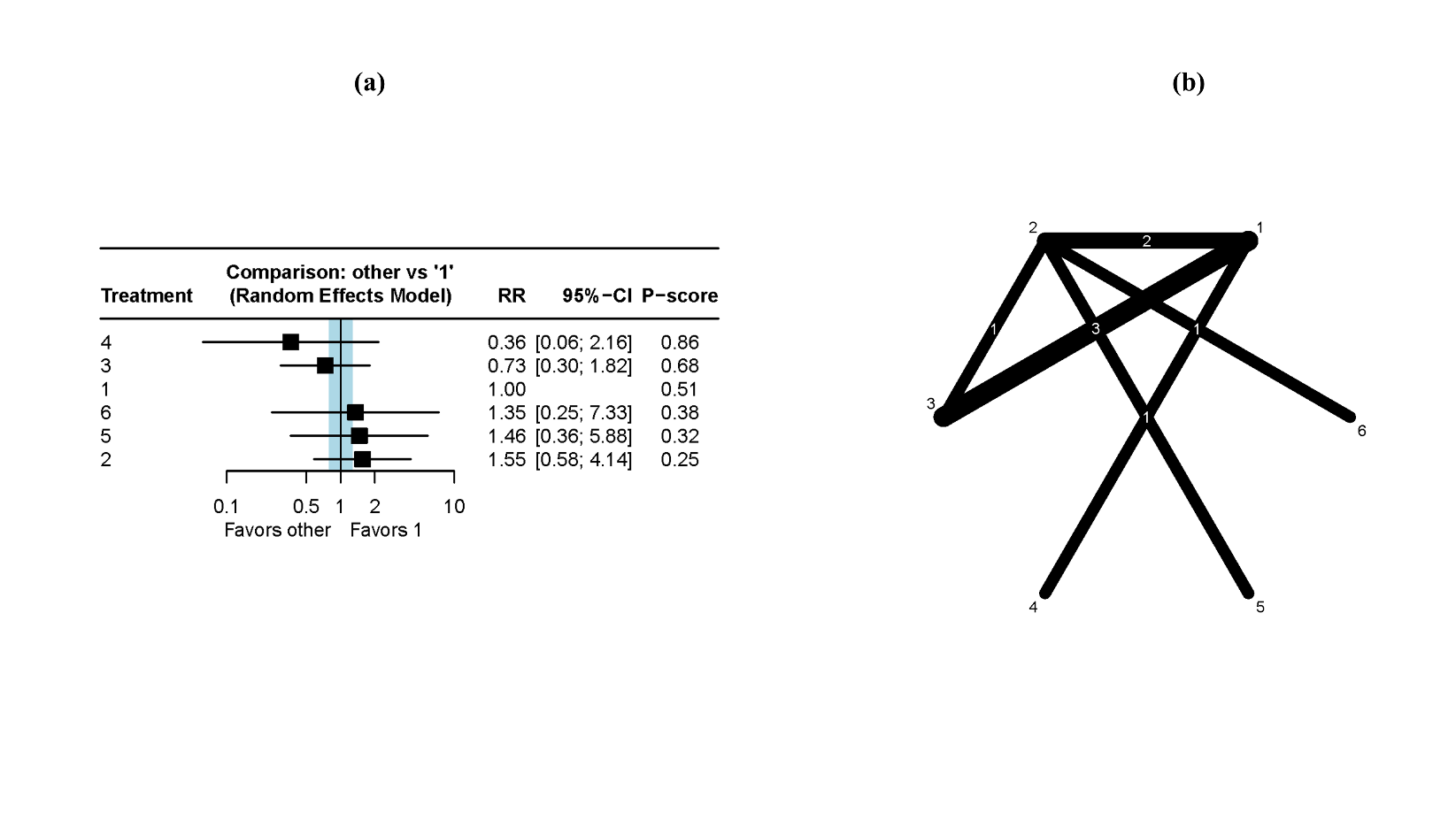

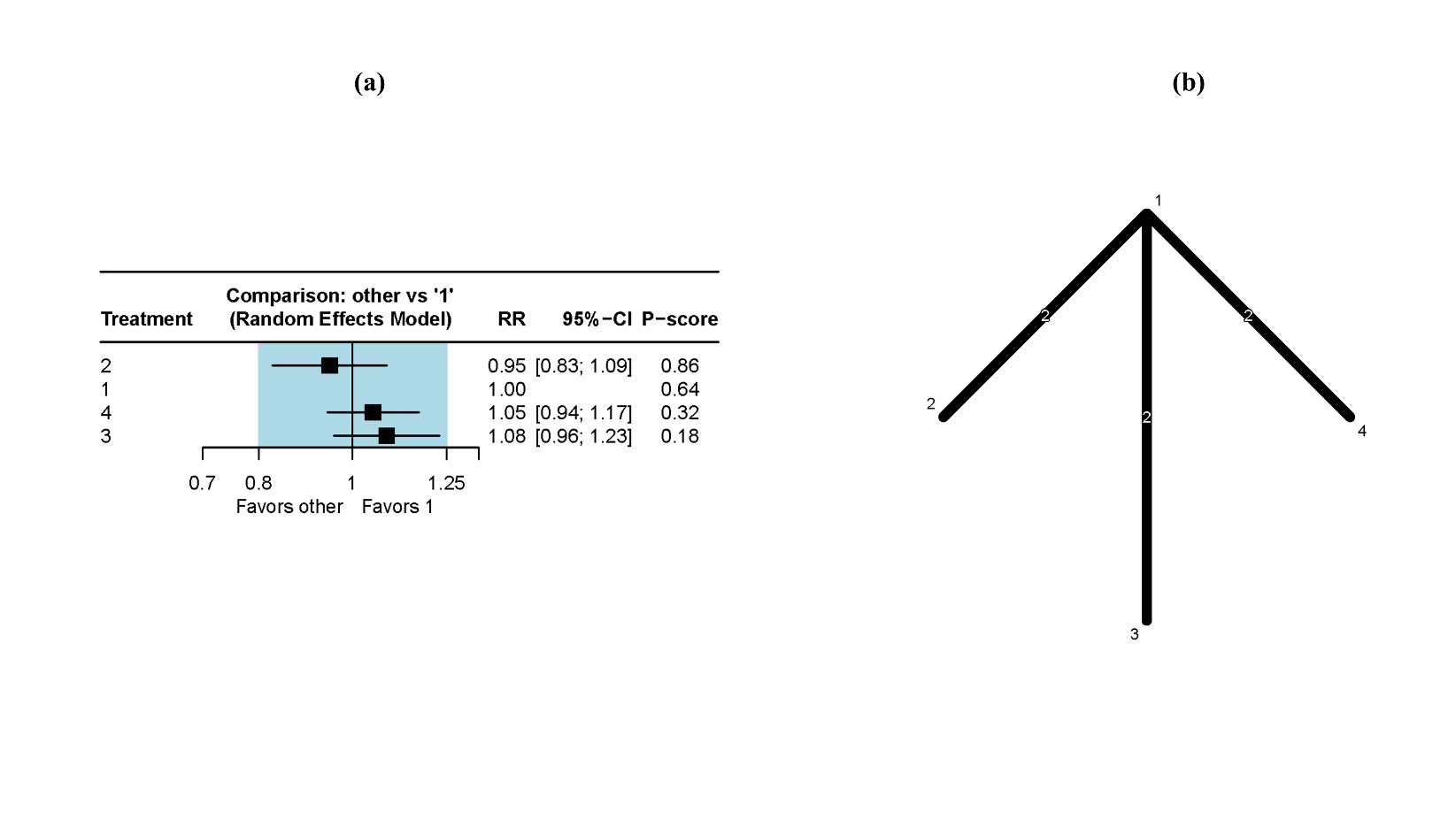


**Figure 9:** Results from the **seventh** of the 21 networks where only ties were identified. Panel (a) presents the NMA estimates and ranking in terms of P-scores, while Panel (b) shows the corresponding network graph. The blue area represents the range of equivalence.

**Figure 8:** Results from the **sixth** of the 21 networks where only ties were identified. Panel (a) presents the NMA estimates and ranking in terms of P-scores, while Panel (b) shows the corresponding network graph. The blue area represents the range of equivalence.


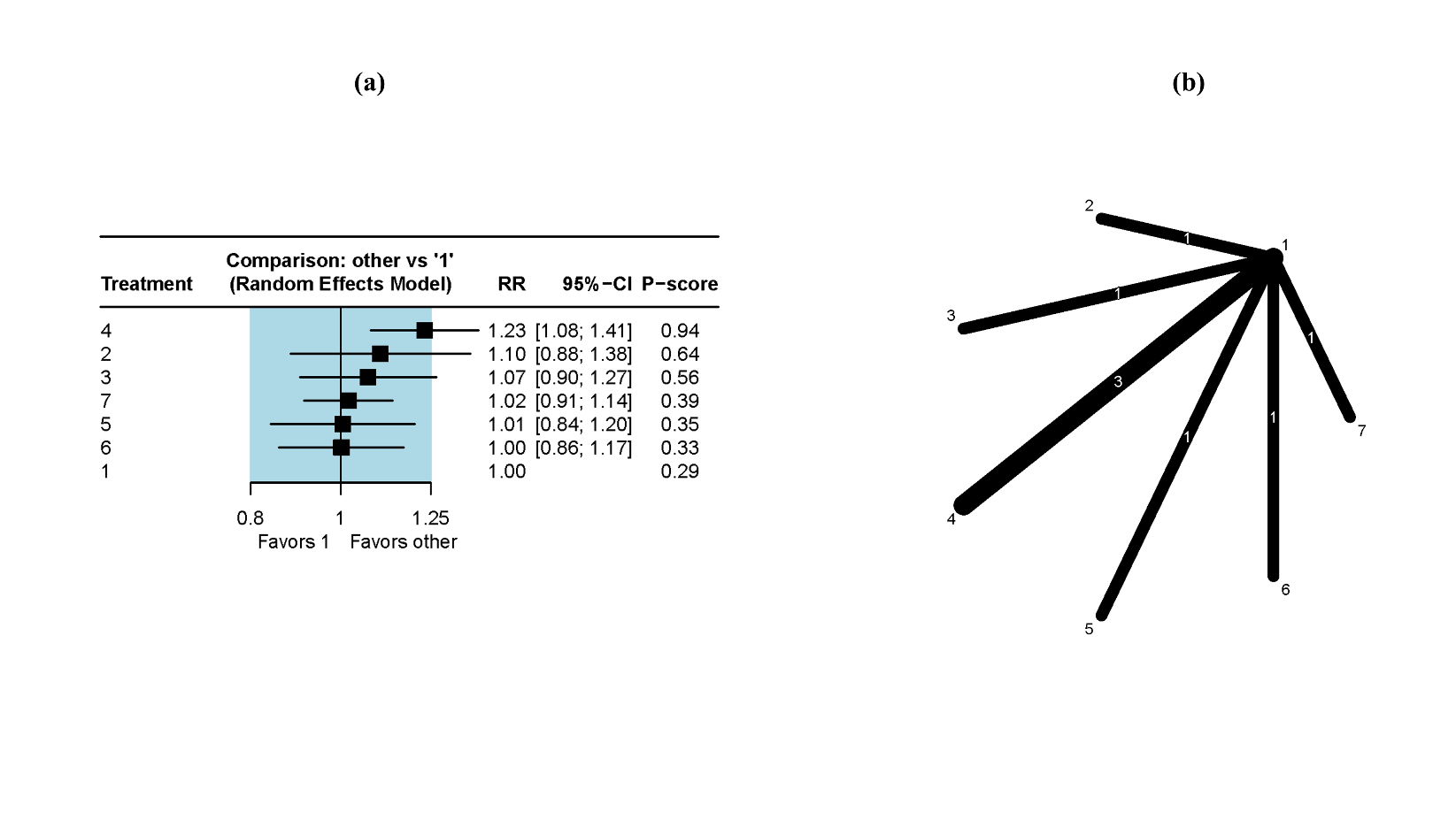

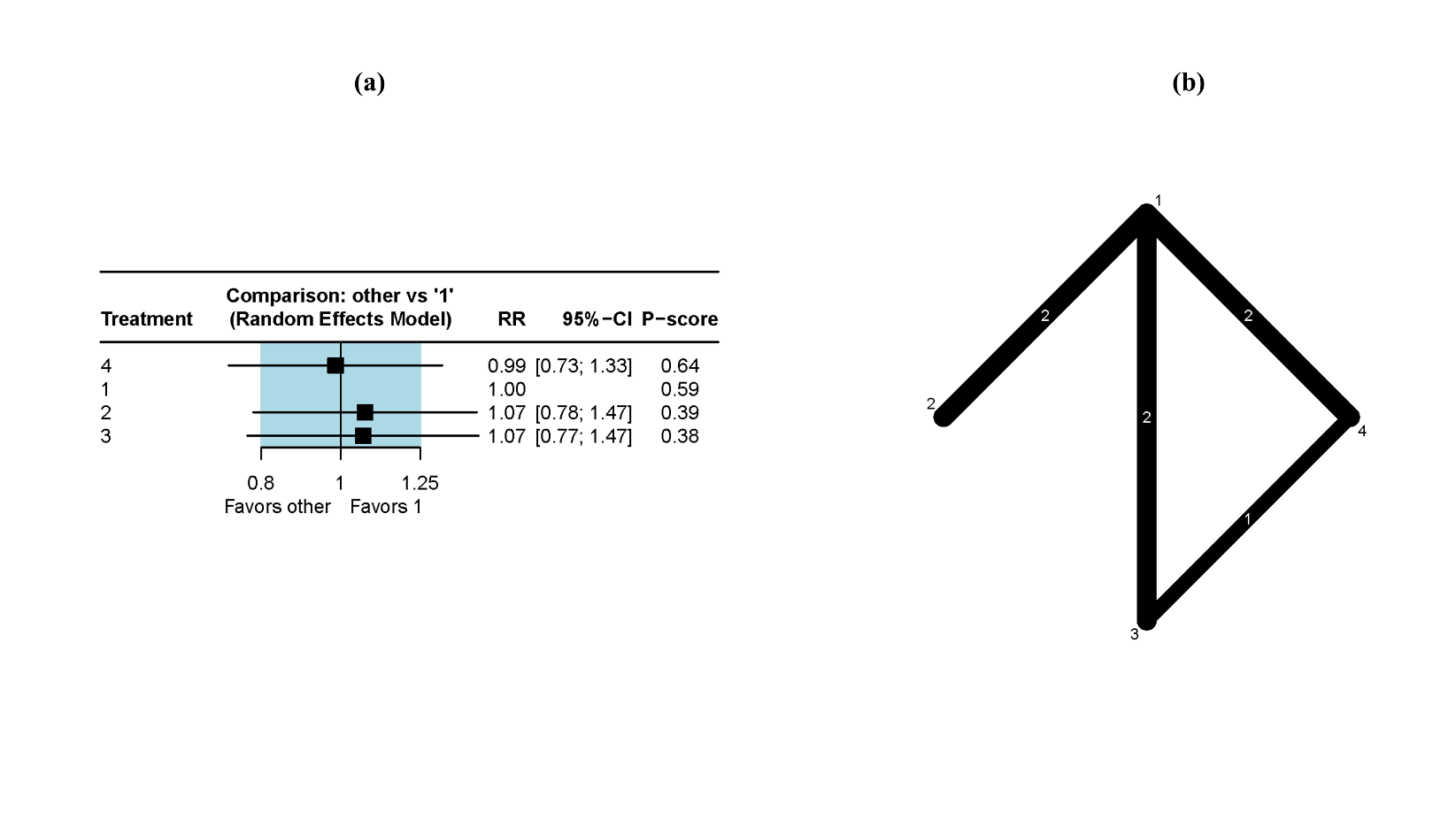


**Figure 10:** Results from the **eigth** of the 21 networks where only ties were identified. Panel (a) presents the NMA estimates and ranking in terms of P-scores, while Panel (b) shows the corresponding network graph. The blue area represents the range of equivalence.

**Figure 11:** Results from the **ninth** of the 21 networks where only ties were identified. Panel (a) presents the NMA estimates and ranking in terms of P-scores, while Panel (b) shows the corresponding network graph. The blue area represents the range of equivalence.


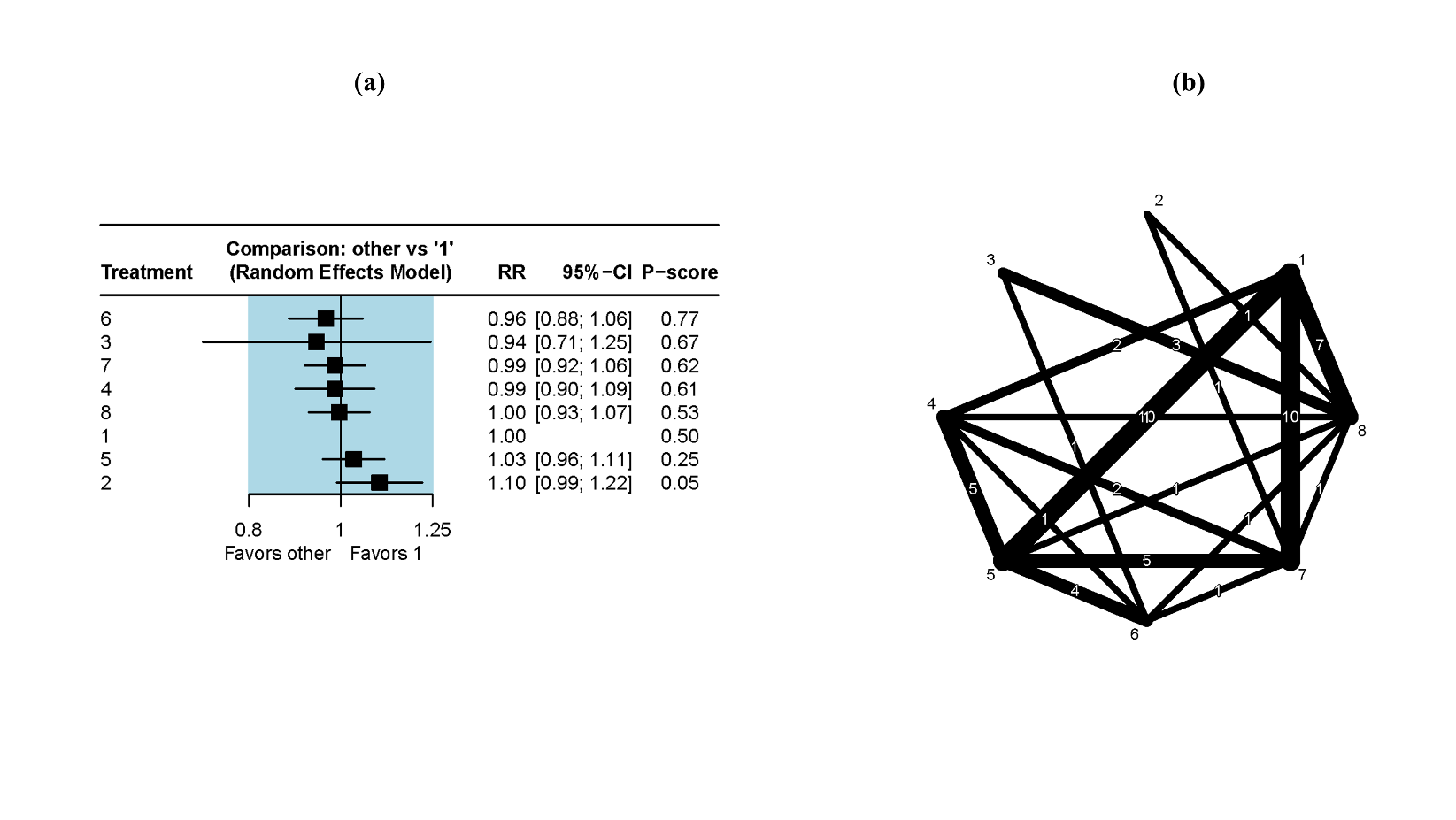

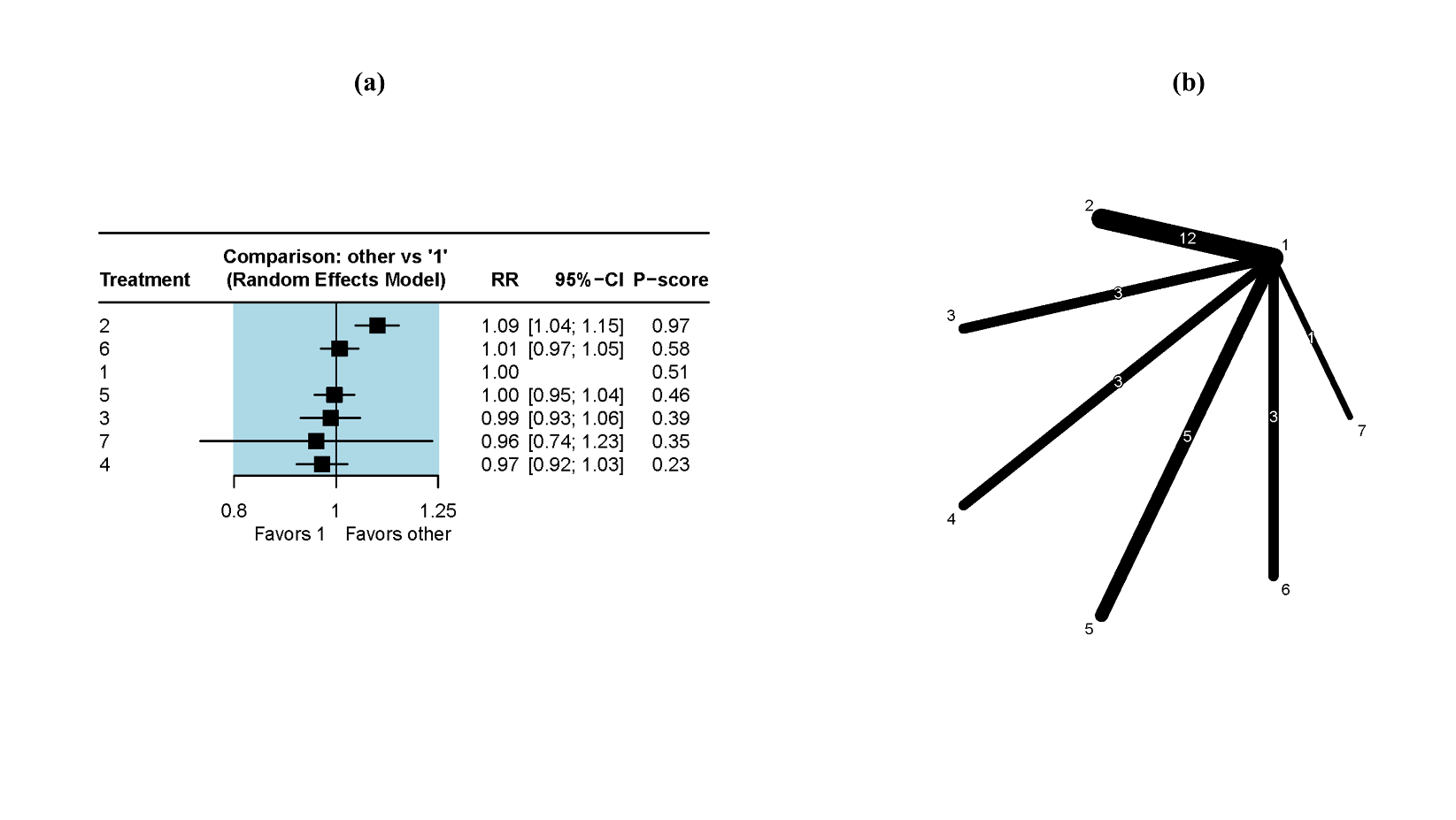


**Figure 13:** Results from the **eleventh** of the 21 networks where only ties were identified. Panel (a) presents the NMA estimates and ranking in terms of P-scores, while Panel (b) shows the corresponding network graph. The blue area represents the range of equivalence.

**Figure 12:** Results from the **tenth** of the 21 networks where only ties were identified. Panel (a) presents the NMA estimates and ranking in terms of P-scores, while Panel (b) shows the corresponding network graph. The blue area represents the range of equivalence.


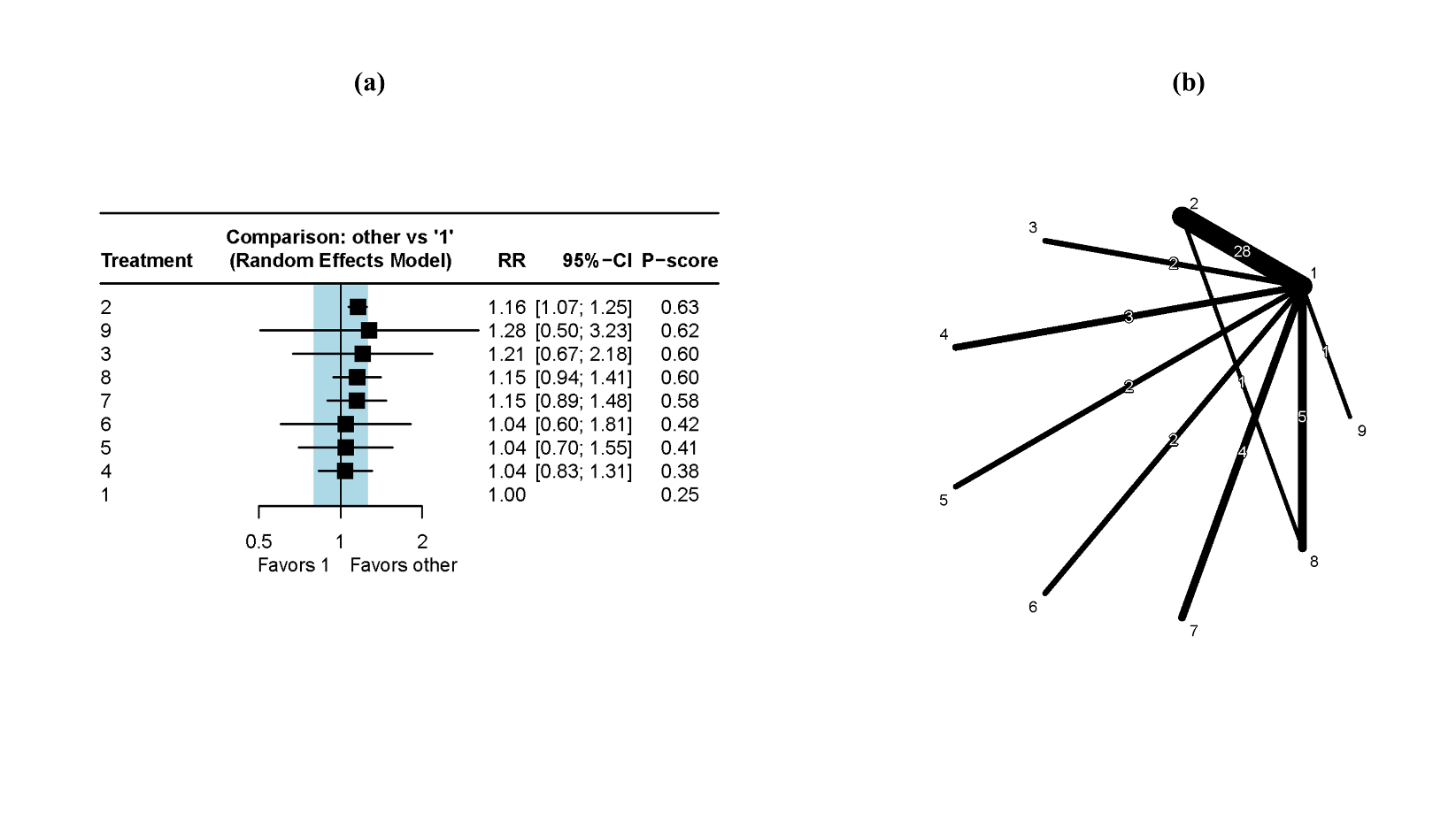

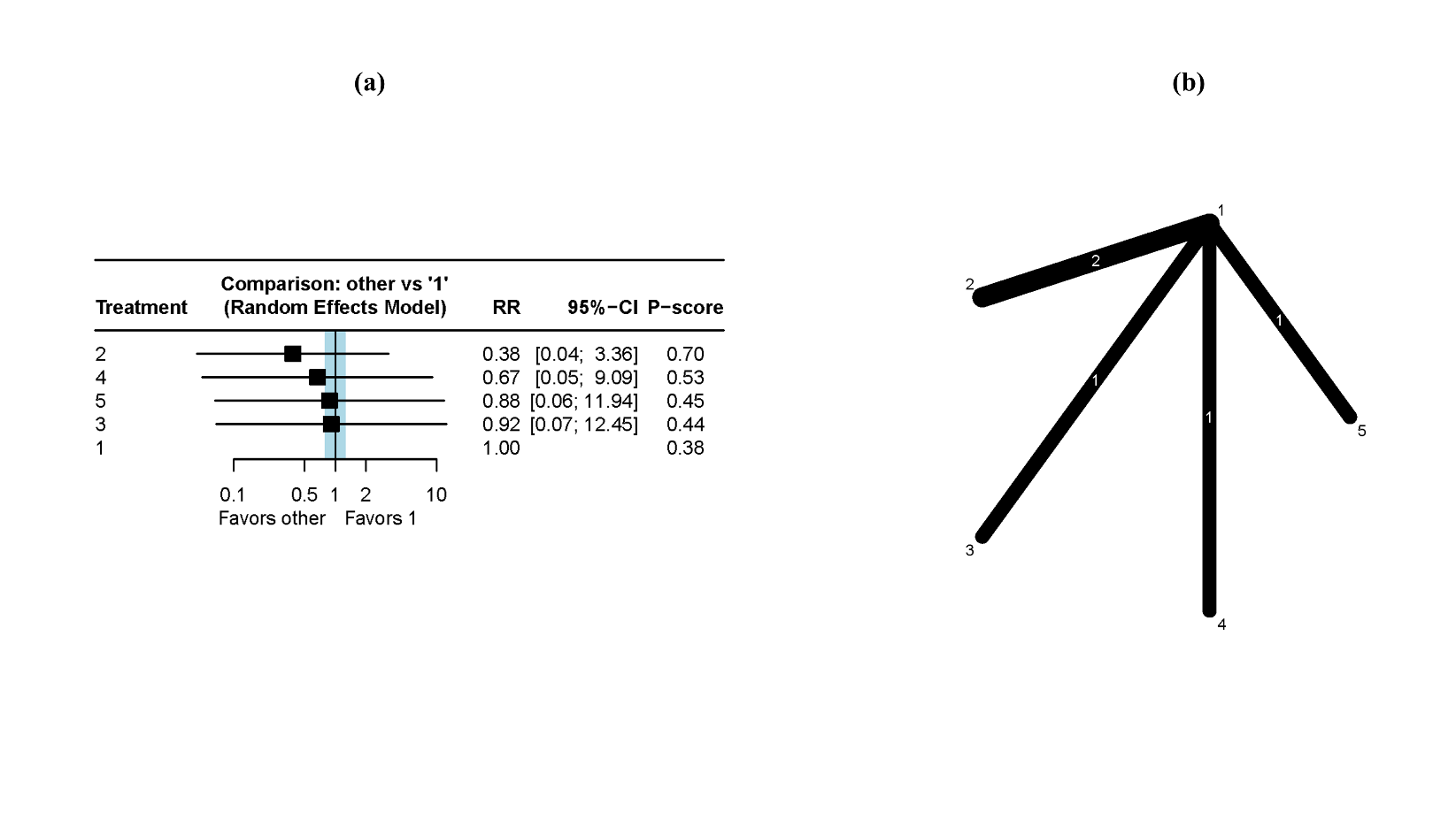


**Figure 15:** Results from the **thirteenth** of the 21 networks where only ties were identified. Panel (a) presents the NMA estimates and ranking in terms of P-scores, while Panel (b) shows the corresponding network graph. The blue area represents the range of equivalence.

**Figure 14:** Results from the **twelfth** of the 21 networks where only ties were identified. Panel (a) presents the NMA estimates and ranking in terms of P-scores, while Panel (b) shows the corresponding network graph. The blue area represents the range of equivalence.


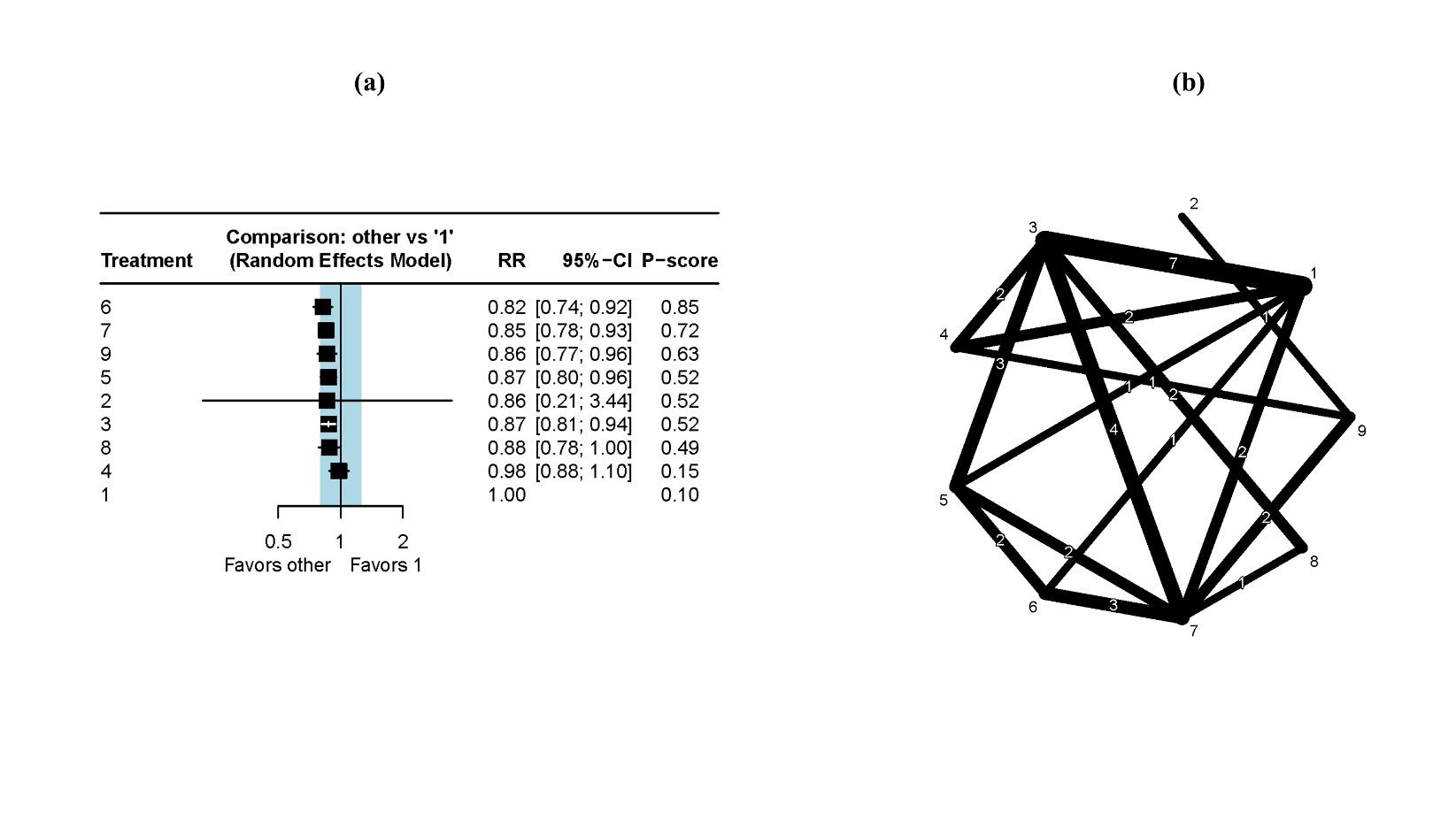

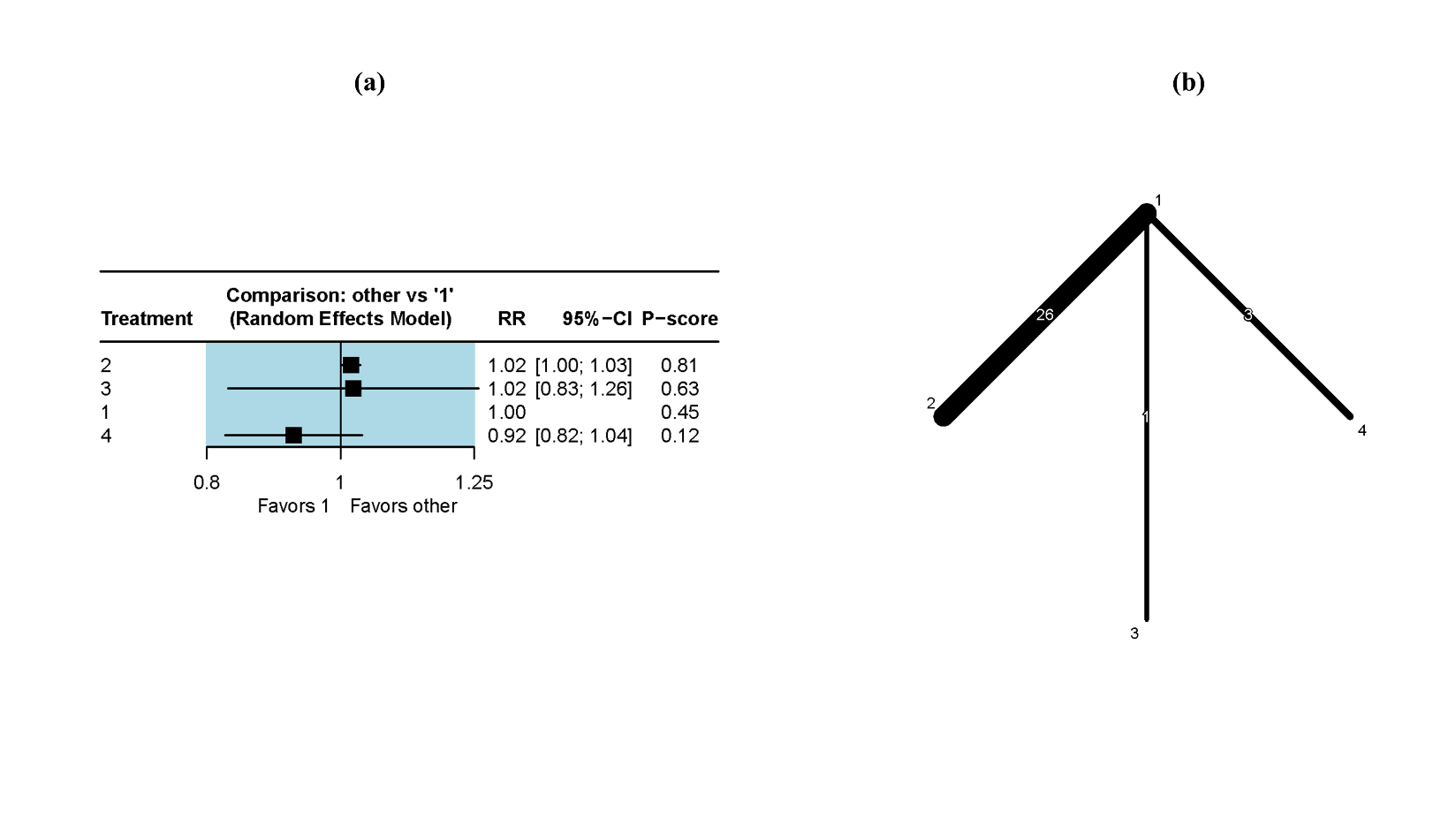


**Figure 17:** Results from the **fiftteenth** of the 21 networks where only ties were identified. Panel (a) presents the NMA estimates and ranking in terms of P-scores, while Panel (b) shows the corresponding network graph. The blue area represents the range of equivalence.

**Figure 16:** Results from the **fourteenth** of the 21 networks where only ties were identified. Panel (a) presents the NMA estimates and ranking in terms of P-scores, while Panel (b) shows the corresponding network graph. The blue area represents the range of equivalence.


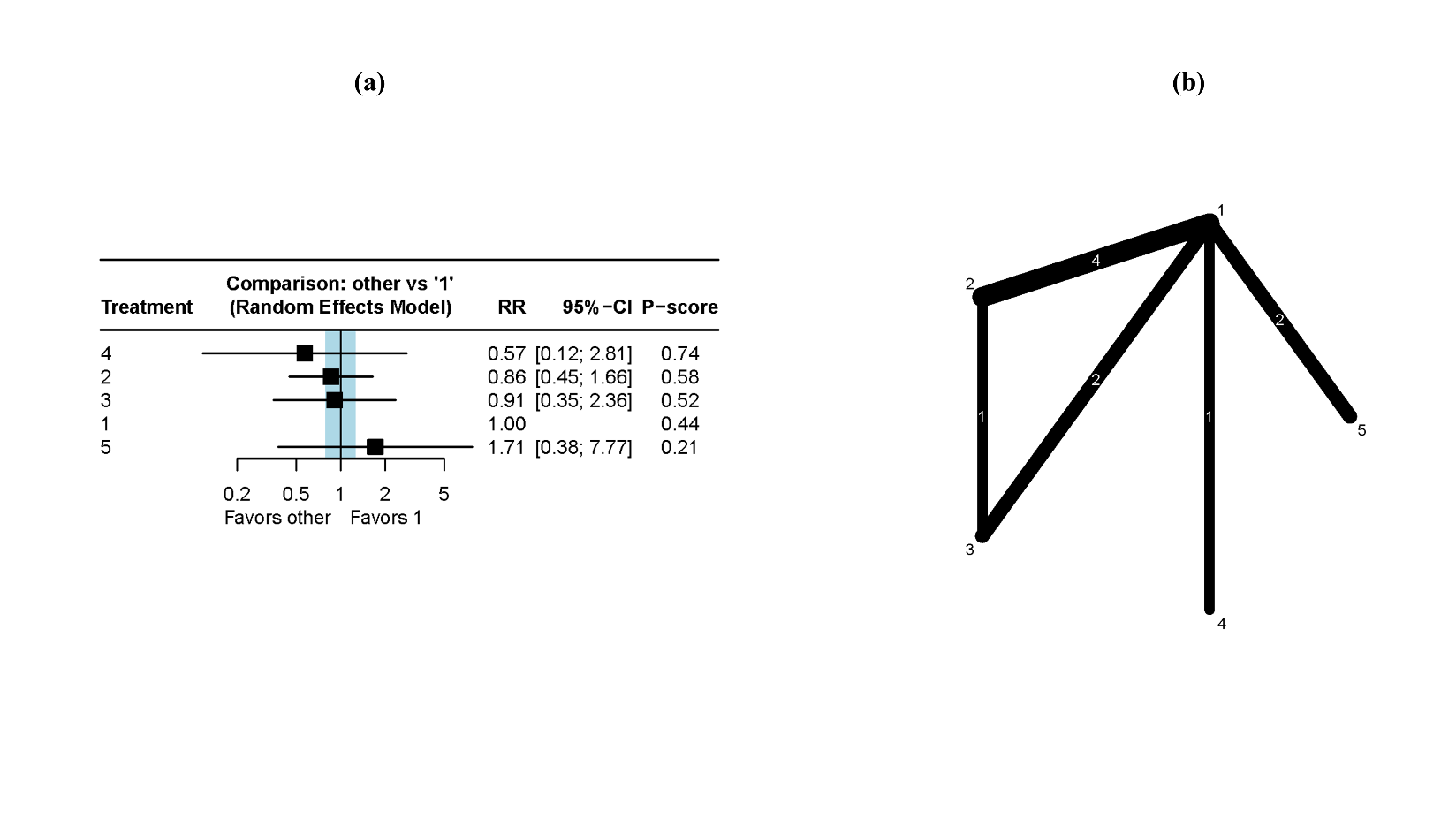

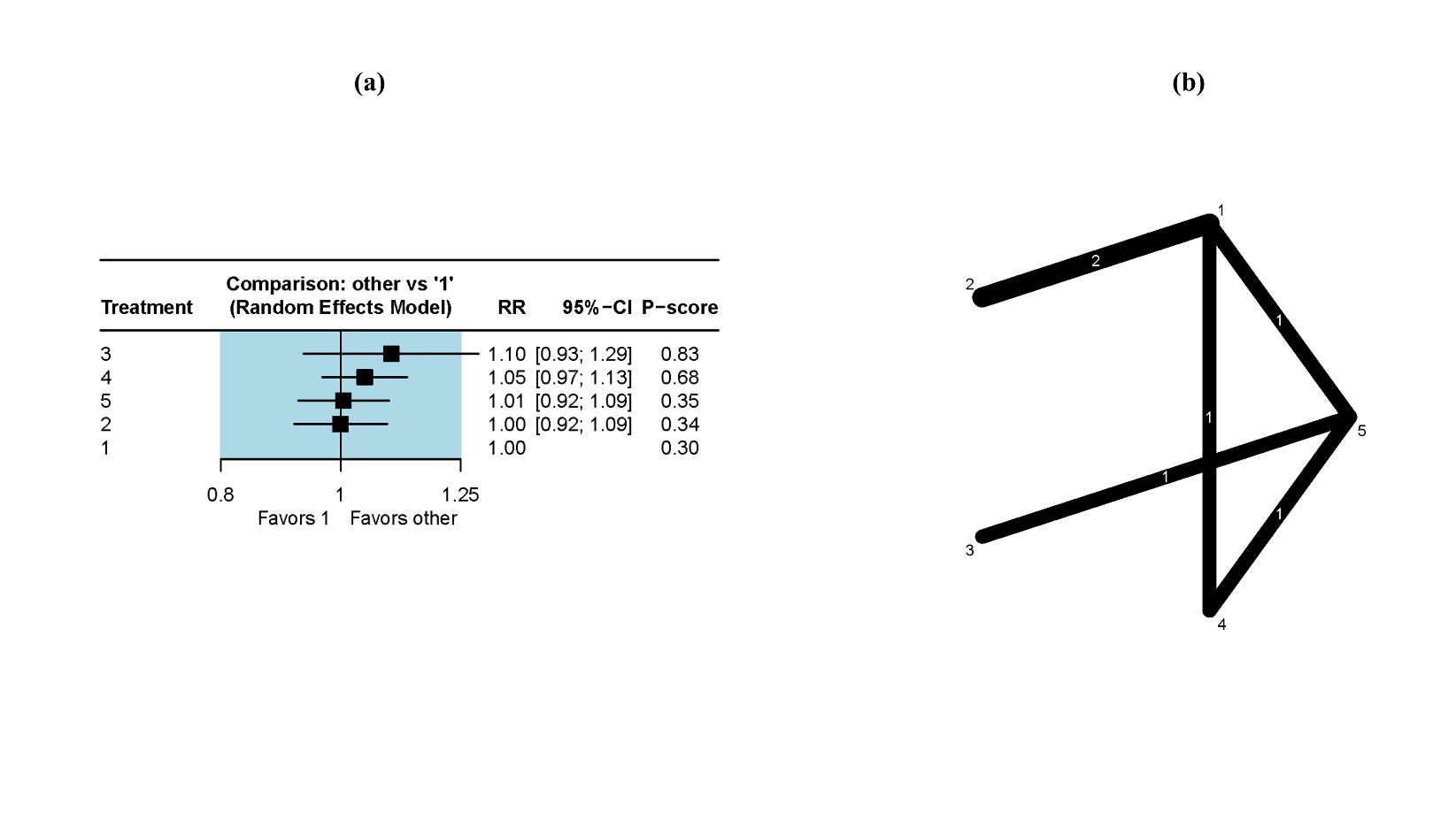


**Figure 19:** Results from the **seventeenth** of the 21 networks where only ties were identified. Panel (a) presents the NMA estimates and ranking in terms of P-scores, while Panel (b) shows the corresponding network graph. The blue area represents the range of equivalence.

**Figure 18:** Results from the **sixteenth** of the 21 networks where only ties were identified. Panel (a) presents the NMA estimates and ranking in terms of P-scores, while Panel (b) shows the corresponding network graph. The blue area represents the range of equivalence.


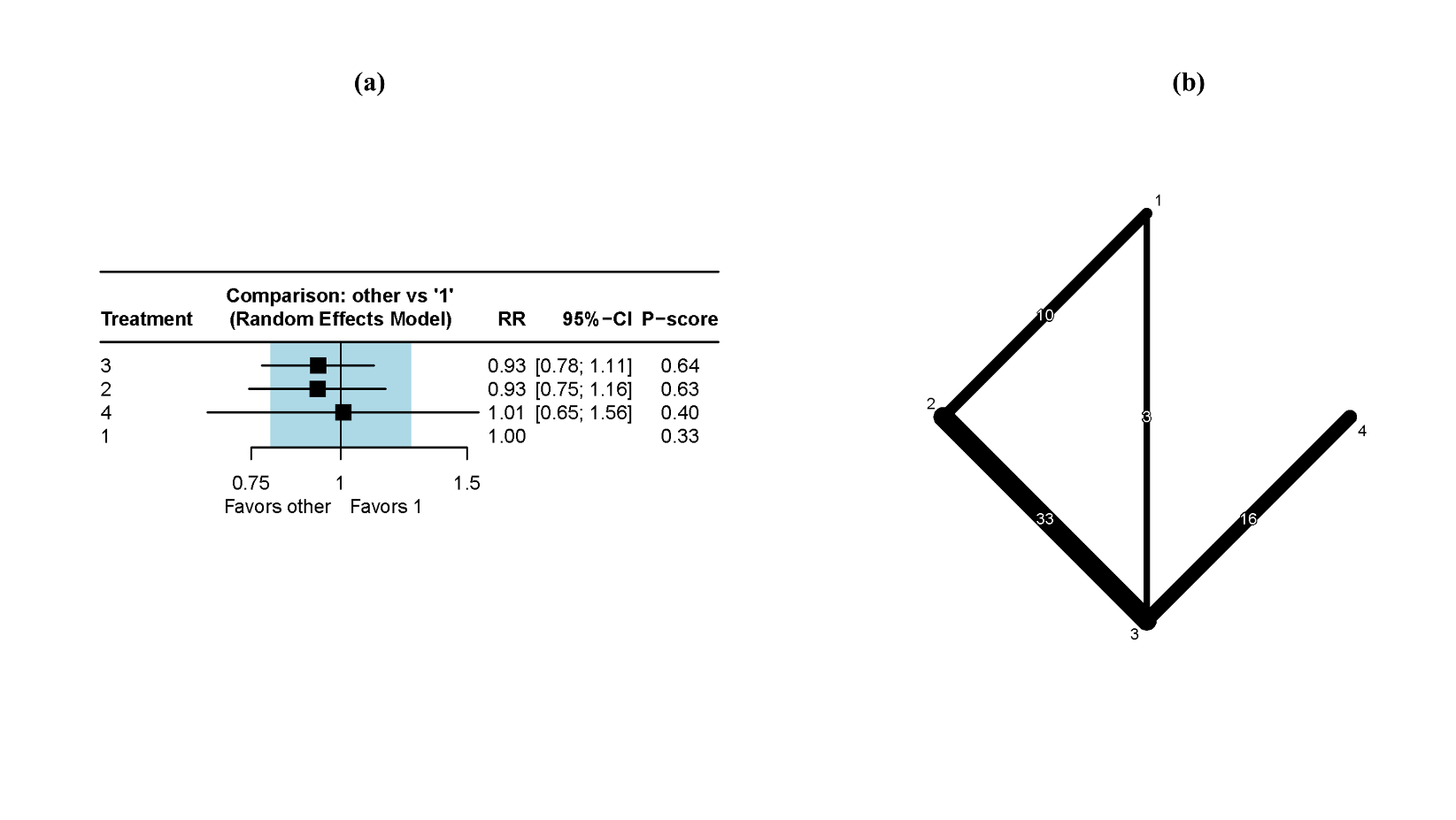

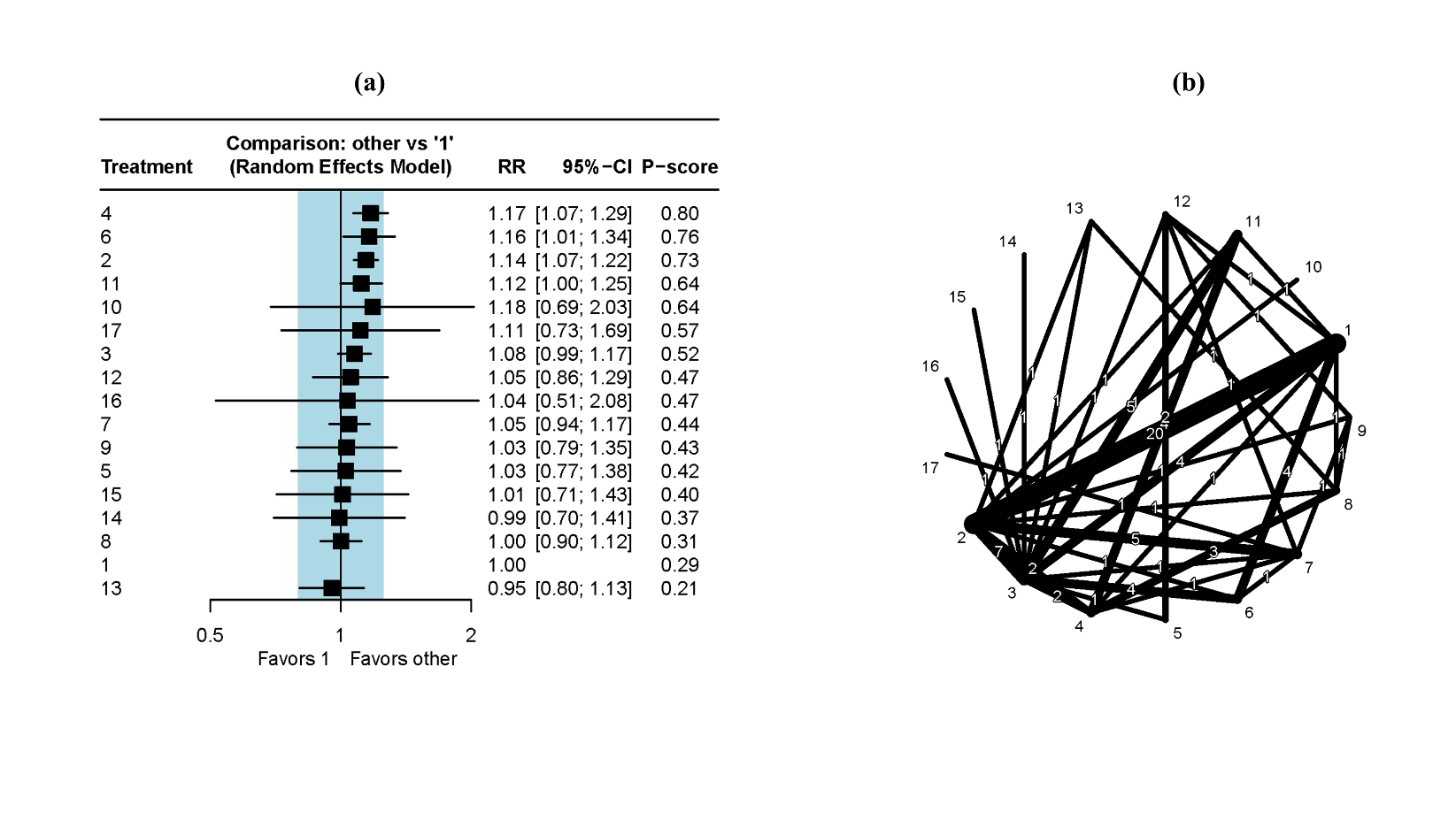


**Figure 20:** Results from the **eighteenth** of the 21 networks where only ties were identified. Panel (a) presents the NMA estimates and ranking in terms of P-scores, while Panel (b) shows the corresponding network graph. The blue area represents the range of equivalence.

**Figure 21:** Results from the **nineteenth** of the 21 networks where only ties were identified. Panel (a) presents the NMA estimates and ranking in terms of P-scores, while Panel (b) shows the corresponding network graph. The blue area represents the range of equivalence.


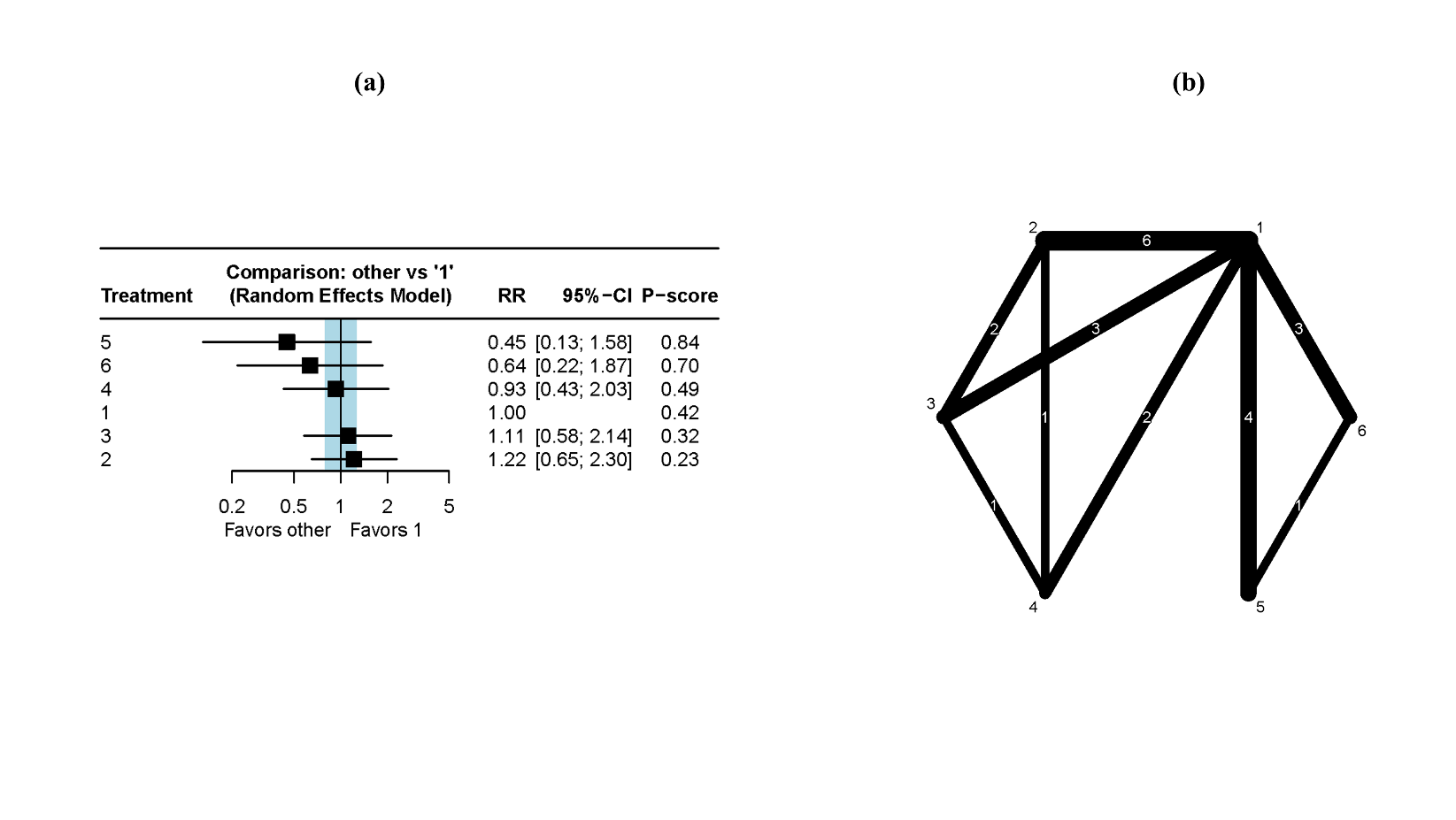

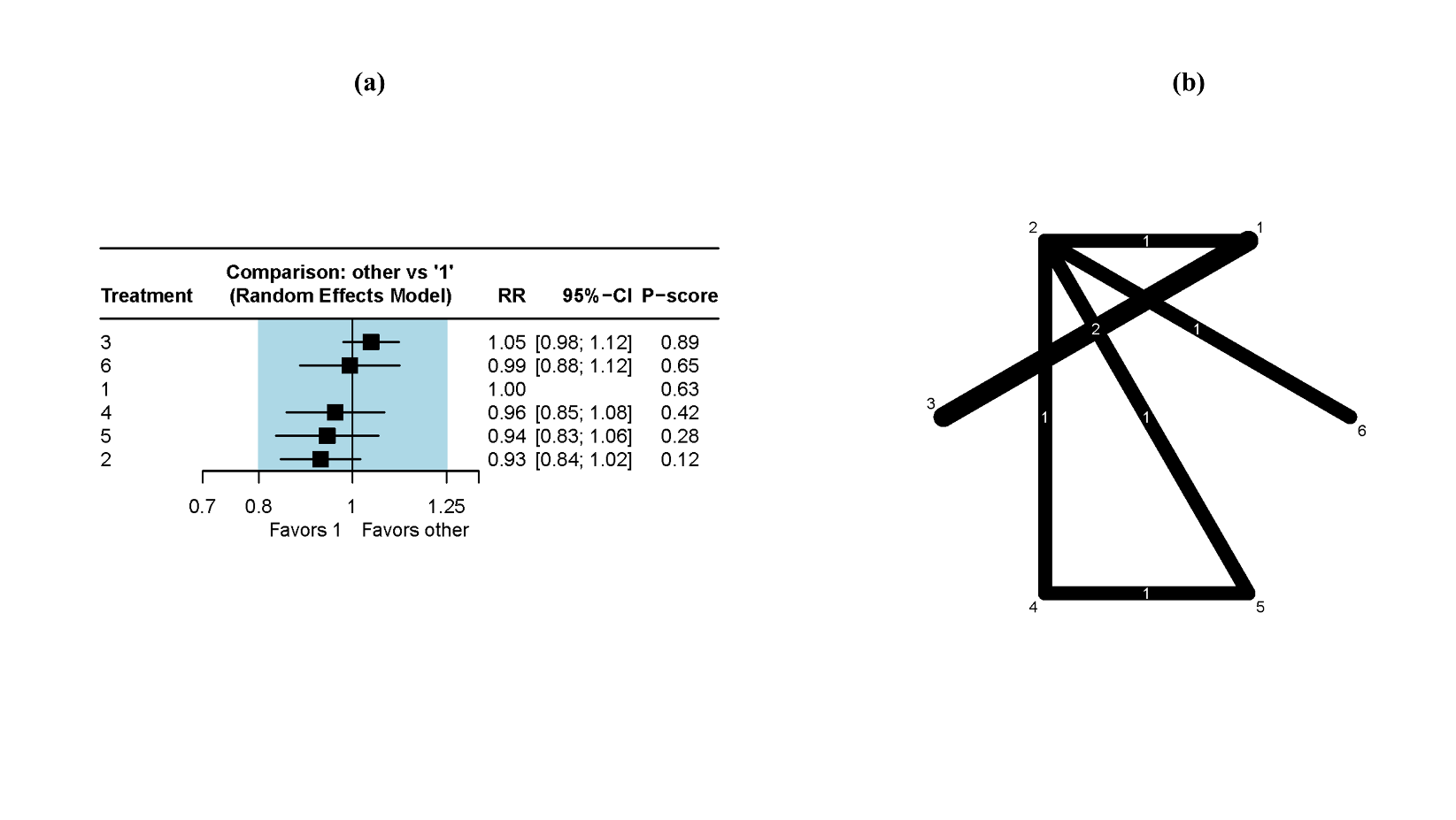


**Figure 23:** Results from the **twenty-first** of the 21 networks where only ties were identified. Panel (a) presents the NMA estimates and ranking in terms of P-scores, while Panel (b) shows the corresponding network graph. The blue area represents the range of equivalence.

**Figure 22:** Results from the **twentieth** of the 21 networks where only ties were identified. Panel (a) presents the NMA estimates and ranking in terms of P-scores, while Panel (b) shows the corresponding network graph. The blue area represents the range of equivalence.
